# Nanostructured Lipid Carriers Enhance Brain Delivery and Antioxidant Efficacy of a Small-Molecule MAO B Inhibitor for Neurodegenerative Disease Therapy

**DOI:** 10.1021/acs.molpharmaceut.5c01754

**Published:** 2026-04-10

**Authors:** Rita Mastrogiacomo, Daniela Valeria Miniero, Mariagrazia Rullo, Federica Rizzi, Gianluca Minervini, Annamaria Panniello, Marinella Striccoli, Elisabetta Fanizza, Roberto Comparelli, Maria Lucia Curri, Marco Catto, Grazia Maria Liuzzi, Leonardo Pisani, Tiziana Latronico, Nicoletta Depalo

**Affiliations:** † Department of Chemistry, 9295 University of Bari Aldo Moro, Via E. Orabona 4, Bari 70126, Italy; ‡ 9327 Institute for Chemical and Physical Processes (IPCF)-CNR SS Bari, Via Orabona 4, Bari 70126, Italy; § Bari Research Unit, National Interuniversity Consortium of Materials Science and Technology (INSTM), 70126 Bari, Italy; ∥ Department Medicine and Surgery, LUM University Giuseppe Degennaro, Casamassima 70010, Italy; ⊥ Department of Pharmacy–Pharmaceutical Sciences, University of Bari Aldo Moro, Bari 70125, Italy; # Department of Biosciences, Biotechnologies and Environment, University of Bari “Aldo Moro”, Bari 70126, Italy

**Keywords:** nanostructured lipid carriers, MAO B inhibitor, blood–brain barrier, neurodegenerative diseases, antioxidant activity

## Abstract

Neurodegenerative disorders, including Alzheimer’s and Parkinson’s diseases, urgently require new therapeutic strategies. Monoamine oxidase B (MAO B), a mitochondrial enzyme involved in oxidative stress and neurotransmitter metabolism, has emerged as a promising target for neuroprotection. A 5-substituted-1*H*-indazole derivative (here referred to as compound **1**) has been recently identified as a potent and safe MAO B inhibitor with antioxidant and neuroprotective properties. Unfortunately, compound **1** suffers from poor aqueous solubility and chemical stability under hydrolytic conditions, thereby limiting its therapeutic potential. To overcome these drawbacks, nanostructured lipid carriers (NLCs) were developed as delivery systems for compound **1**. The coloading of luminescent carbon dots (CDs) together with compound **1** within NLCs further enabled investigation into NLCs’ ability to permeate through the artificial blood–brain barrier (BBB) model, allowing a quantitative evaluation of crossing efficiency. Delivery via NLCs resulted in a markedly higher fraction of compound **1** crossing the BBB (∼26%) compared with the free molecule (∼2.6%). Encapsulation also retained antioxidant efficacy in SH-SY5Y cells, while the nanoformulations exhibited a good degree of cell tolerance, with viability remaining above 60% across the tested concentration range. These in vitro findings suggest that the proposed nanoformulation represents a promising strategy to enhance delivery of the investigated small molecule to the central nervous system (CNS), highlighting its potential application in neurodegenerative diseases (NDs).

## Graphical abstract

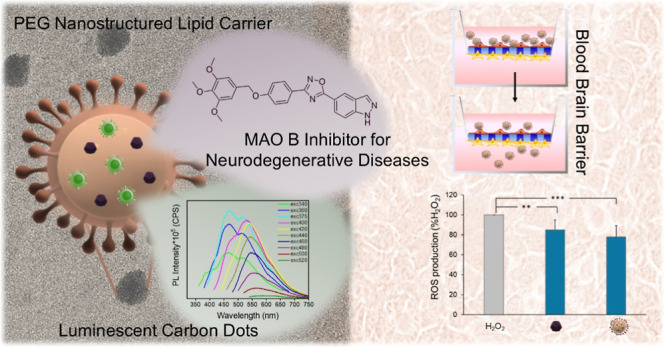

## Introduction

The increased life expectancy boosts the prevalence of age-related disorders, including Alzheimer’s disease (AD) and Parkinson’s disease (PD). These pathologies are progressive and debilitating neurodegenerative disorders (NDs) characterized by cognitive decline, behavioral disturbances, and memory impairment, ultimately compromising patients’ ability to perform daily activities. The growing socioeconomic burden is due to the paucity of reliable early diagnostic tools as well as the lack of effective disease-modifying therapies. Each ND presents disease-specific histopathological hallmarks. For example, those of AD include the accumulation of extracellular neuritic plaques, predominantly composed of β-amyloid (Aβ) fibrils, and intracellular neurofibrillary tangles formed by aggregates of hyperphosphorylated tau protein within neurons.^1^ In addition to these hallmarks, a growing body of evidence implicates several interconnected pathological mechanisms in NDs, including synaptic dysfunction, chronic neuroinflammation, mitochondrial derangement, decline of bioenergetic metabolism, oxidative stress, and vascular abnormalities.^2^ On these bases, NDs are referred to as multifactorial diseases where the simultaneous presence of several abnormalities has made it quite hard to establish downstream triggering events and validate effective drug targets. Given the multifactorial nature of NDs, one of the major challenges in drug development is the need for a paradigm shift from single-target approaches to strategies capable of addressing the complexity of the disease. In this context, the multitarget-directed ligand (MTDL) approach has emerged as a promising strategy, aiming at designing hybrid small molecules capable of simultaneously modulating multiple key pathological pathways implicated in AD onset and progression.^3–10^ This strategy offers therapeutic advantages by leveraging the synergistic effects of multifunctional activity, minimizing drug–drug interactions, simplifying treatment regimens, and ultimately improving safety, efficacy, and patient compliance. On the other hand, the complexity of these diseases requires the identification of novel druggable targets. In this context, mitochondrial membrane-bound enzymes^4,11^ named monoamine oxidases A and B (MAO A and B) are worth noting. These flavoenzymes are responsible for the catabolic degradation of endogenous (e.g., catecholamines) and exogenous monoamines by means of oxidative deamination reactions. Apart from neurotransmitter modulation enabled by MAO inhibitors, these enzymes represent promising targets to provide neuroprotective activities against NDs. The catalytic action of MAO produces hydrogen peroxide as a harmful byproduct, contributing to the propagation of reactive oxygen species (ROS) and leading to oxidative damage in mitochondria and cells. MAO B, in particular, elicits active roles in neuroinflammation, sustaining the activation of NLRP3 (NLR family pyrin domain containing 3) inflammasome, also responsible for neurodegeneration.^12^ Increasing evidence suggests that neuroinflammation, especially involving microglia and astrocytes, is a prominent feature in the early stages of AD.^13^ Reactive astrocytes release pro-inflammatory cytokines and overexpress MAO B.^14^ Furthermore, in AD brains, MAO B colocalizes with amyloid plaques,^15^ and its neuronal staining is associated with Aβ_42_ production.^16^ Silencing MAO B has been shown to enable Aβ_42_ depletion, suggesting that the blockade of MAO B activity may contribute to mitigating the neuroinflammation in AD. In this context, a series of 5-substituted-1*H*-indazoles acting as neuroprotective MAO B inhibitors have been discovered through a molecular hybridization strategy developed by some of the present authors.^17^ After the evaluation of inherent cytotoxicity against human neuroblastoma cell line SH-SY5Y, the safest compounds were tested for their ability to counteract the cytotoxic damage and ROS production on the SH-SY5Y line triggered insulted by hydrogen peroxide.^18^ Among all the proposed molecules, the compound hereafter referred to as compound **1** (indazole 20 in the original paper) emerged as a promising candidate in terms of enzymatic inhibition, antioxidant activity, and neuroprotective properties. However, its further development was hampered by suboptimal drug-likeness, mainly influenced by physicochemical limitations, primarily due to critical aqueous solubility.^18^ These inappropriate features often represent the bottleneck of central nervous system (CNS) drug discovery programs as inadequate drug-like properties are the main reason for low bioavailability and poor brain penetration. To address these issues and enhance the therapeutic potential of this small molecule, nanostructured lipid carriers (NLCs) have been investigated here as delivery systems for compound **1**. This lipid-based nanoplatform has been selected to improve its permeability across the blood–brain barrier (BBB), increase bioavailability, and enhance the antioxidant efficacy. NLCs have gained significant attention in the field of neuropharmacology as efficient brain-targeted drug delivery systems, offering benefits such as controlled release profiles, low systemic toxicity, and high colloidal stability, facilitating the transport of therapeutic agents across the BBB.^19–25^ Their solid lipid matrix provides protection against enzymatic and hydrolytic degradation of the therapeutic cargo, prolonged circulation time, and reduced clearance rates.^26^ Moreover, the lipidic nature of NLCs facilitates transport across the BBB even in the absence of surface functionalization. Although the exact mechanism by which lipid-based nanoparticles cross the BBB is unclear, lipidic nanostructures with dimensions ranging from 40–200 nm can cross the tight BBB endothelial cells, avoiding the rapid uptake by the reticuloendothelial system (RES).^20^ Supporting this strategy, previous studies, such as that of Cunha et al.,^27^ successfully employed NLCs to enhance the BBB permeability of small therapeutic agents like rivastigmine for AD treatment.

In the present study, NLCs loaded with compound **1** have been prepared via a hot homogenization-evaporation microemulsion technique and characterized in terms of particle size, morphology, colloidal stability, and encapsulation efficiency. The resulting formulations have been subjected to in vitro evaluation to assess their cytotoxicity and antioxidant efficiency as well as their ability to deliver the small molecule across an artificial BBB model. To enable monitoring of BBB crossing, oil-dispersible Carbon Dots (CDs) have been synthesized and coloaded with compound **1**, yielding an in vitro optically traceable formulation.

## Materials and Methods

### Materials

Citric acid (anhydrous), 1-octadecene (ODE, technical grade 90%), 1-hexadecylamine (HDA, 98%), Kolliphor P188, cholesterol, cholesteryl oleate, glyceryl trioleate, 3-(4,5-dimethylthiazol-2-yl)-2.5- diphenyltetrazolium bromide (MTT), fluorescein isothiocyanate-dextran (FITC-D, average molecular weight: 3000–5000), hydrogen peroxide solution 30% stabilized, 2-(N-morpholino)­ethanesulfonic acid, (MES), sodium chloride (NaCl), disodium hydrogen phosphate (Na_2_HPO_4_), potassium chloride (KCl), potassium dihydrogen phosphate (KH_2_PO_4_), human recombinant Monoamine Oxidase B (MAO B), and safinamide were purchased from Sigma-Aldrich, St. Louis, MO. Additionally, 1, 2-distearoyl-*sn*-glycero-3-phosphoethanolamine-poly­(ethylene glycol) (DSPE- PEG) was obtained from Avanti Polar Lipids. Fetal bovine serum (FBS), penicillin, and streptomycin (P/S) were provided by GIBCO (Paisley, Scotland), while Dulbecco’s modified Eagle’s medium (DMEM) was obtained from SIAL, Roma, Italy. 2′,7′-Dichlorodihydrofluorescein diacetate (DCFH-DA) was purchased from Calbiochem, San Diego, CA, USA. Rat Tail Collagen I (high concentration) and Transwell cell culture inserts were from Corning (New York).

The astrocyte DI TNC1 cell line (ATCC CRL-2005 https://www.atcc.org/products/crl-2005), human neuroblastoma cells (SH-SY5Y, ATCC CRL-2266 https://www.atcc.org/products/crl-2266), and brain immortalized endothelial cell line (bEnd.3, ATCC CRL-2299 https://www.atcc.org/products/crl-2299) were obtained from the American Type Culture Collection (ATCC). According to the supplier’s quality control reports, the cell lines were tested and confirmed to be free of mycoplasma contamination at the time of purchase.

All chemicals and reagents used were of analytical grade. Ultrapure water (resistivity: 18.2 MΩ·cm; total organic carbon ≥4 μg/L) was obtained using a Milli-Q Gradient A-10 system (Millipore) and utilized for the preparation of all aqueous solutions.

### Compound **1** Synthesis

The medium-scale synthesis (starting from 0.50 g of 1*H*-indazole-5-carboxylic acid, replicated as required, overall yield 23 ± 2%) of compound **1** was performed according to an already published procedure, where the interested reader can also find the details of materials, methods, analytical, and spectroscopic characterization.^17^ Briefly, the Mitsunobu-type alkylation of 4-cyanophenol with (3,4,5-trimethoxyphenyl)­methanol afforded the desired nitrile, which was further transformed into the corresponding amidoxime by reacting with hydroxylamine. Then, pyridine under reflux conditions promoted the cyclization of 1*H*-indazole-5-carboxylic acid, activated in situ as acyl chloride, with the amidoxime to the desired 1,2,4-oxadiazole **1** (Scheme 1).

**Scheme 1:**
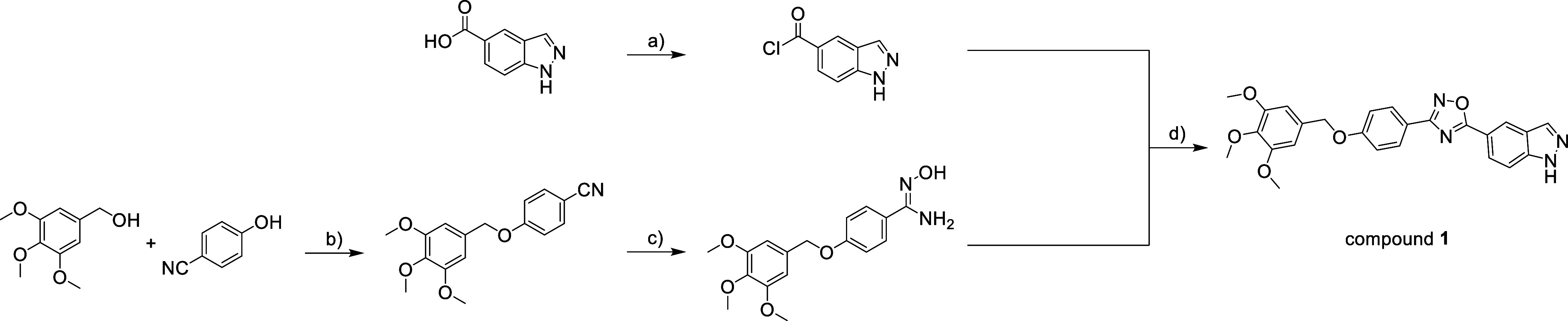
^a^

a Reagents and conditions: (a) thionyl chloride, anhydrous dichloromethane, Δ, 4 h; (b) triphenylphosphine, di-isopropyl azodicarboxylate, anhydrous tetrahydrofuran, room temperature, 72 h; (c) hydroxylamine 50% wt. in water, ethanol, room temperature, 72 h; (d) anhydrous pyridine, Δ, 5 h.

White solid; mp 189–192 °C. ^1^H NMR and ^13^C NMR spectra and high-resolution mass spectra were in full agreement with those already reported.^17^ The purity of newly prepared batches was always checked by elemental analysis and RP-HPLC (>97%). The percentage values found for C, H, and N agreed to within ±0.40% of the theoretical values. The purity of compound **1** used for all biological experiments was >97% (by elemental analysis and RP-HPLC, see Supporting Information, Figure S1).

### Preparation of NLC-Based Formulations

Empty NLCs were prepared by the hot homogenization-evaporation technique according to previously reported procedures^28,29^ with some modifications. Cholesterol, cholesteryl oleate, glyceryl trioleate, and DSPE-PEG (1:1.64:0.24:0.32 lipids ratio w/w) were dissolved in chloroform (792.4 μL). The organic solution was poured into a Kolliphor P188 aqueous solution (10 mL, 1.18 mg) and maintained at 50 °C, and then, the mixture was homogenized with an ULTRA-TURRAX homogenizer for 5 min at 20,000 rpm. The resulting oil-in-water emulsion was left 1 h at 35 °C, 1 h at 25 °C, and then overnight at room temperature to let chloroform evaporate. Samples were stored at 4 °C.

NLCs loaded with compound **1** (**1**/NLCs) were prepared using 795 μL from chloroform solutions of compound **1** at different concentrations (1, 2, and 3 mg/mL) instead of pure chloroform.

To obtain optically traceable nanovectors (**1**-CD/NLCs), luminescent CDs were synthesized according to the experimental procedure reported by Panniello et al.,^30^ based on the thermal decomposition of citric acid in a high-boiling solvent system. Specifically, the reaction medium was ODE, while HDA acted simultaneously as a surface-capping ligand and nitrogen dopant source. The reaction was conducted under nitrogen flow using Schlenk techniques and anhydrous or degassed reagents. HDA (6.2 mmol) was first solubilized in ODE (26 mL) at 100 °C under vacuum for 30 min. Subsequently, the reaction batch was further heated, and 5.2 mmol of citric acid was quickly injected into the hot reaction mixture at 200 °C. After maintaining these conditions for 3 h, the reaction was stopped by cooling down the system to 25 °C. The crude product was subjected to multiple precipitation–redispersion cycles using acetone as the nonsolvent, yielding purified C-dots that were ultimately redispersed in chloroform (620 mg/mL).

For the preparation of optically traceable NLCs coencapsulating CDs and compound **1** (**1**-CD/NLCs), 8 μL of a CD dispersion in chloroform (620 mg/mL) was added to the organic phase during nanoformulation preparation.

For all the preparations, after synthesis, samples were concentrated to a final volume of 2 mL and then washed with water to remove the excess lipids, nonembedded compound **1**, and/or CDs by centrifugation cycles at 4 °C (Centricon centrifugal filter).

### Characterization of the NLC-Based Formulations in Terms of Size, Morphology, Colloidal Stability, and Optical Properties

The hydrodynamic diameter, size distribution, polydispersity index (PDI), and colloidal stability of the different NLC samples were evaluated by using a Zetasizer NanoZS, Malvern Instruments Ltd., Worcestershire, U.K. (DTS 5.00) (sample dilution 1:100 in ultrafiltered water). All reported data are presented as mean values ±standard deviation (SD) (three replicates). Transmission Electron Microscopy (TEM) investigation was performed using a JEOL JEM-1011 microscope working at an accelerating voltage of 100 kV, and TEM images were acquired by an Olympus Quemesa Camera (11 Mpx). The samples were prepared by dropping an aqueous suspension of NLCs on a 400 mesh amorphous carbon-coated Cu grid and letting the solvent evaporate.

UV–vis absorption measurements were performed with a Varian Cary 5000 UV–vis–NIR double-beam spectrophotometer. Attenuated total reflection Fourier transform infrared (ATR-FTIR) spectra of samples were recorded by using a PerkinElmer Spectrum One Fourier Transform Infrared spectrometer equipped with a 4 mm diameter diamond microprism as an internal reflection element. All the spectra were recorded in the range of 400–4000 cm^–1^ with a resolution of 4 cm^–1^ (16 scans). The steady-state photoluminescence (PL) emission spectra of the CD-loaded samples were recorded by using a Fluorolog 3 spectrofluorometer (Horiba Jobin-Yvon) equipped with a 450 W Xe lamp as the excitation light source, coupled with double grating excitation and emission monochromators. Absolute Quantum Yield (QY) measurements were obtained by means of a “Quanta-phi” integrating sphere mounted in the optical path of the Fluorolog 3 spectrofluorometer (Horiba Jobin-Yvon). Time-resolved (TR)-PL measurements were performed by a time-correlated single photon counting (TCSPC) technique, with a FluoroHub (Horiba Jobin-Yvon). The samples were excited at 375 nm by a picosecond laser diode (NanoLED 375L). PL signals were detected by a picosecond photon counter (TBX ps Photon Detection Module, Horiba Jobin-Yvon). The time resolution of the experimental setup was set to ≈200 ps. The decay function was determined from the output histogram by performing a best-fitting procedure using a multiexponential model by DAS Analysis software, which allowed us to derive the average lifetime (τ_avg_). The goodness of fit was assessed by the *X* ^2^ parameter (as usually accepted, only fitted curves with *X* ^2^ < 1.2 were considered), minimizing the residual plot.

### Evaluation of the Encapsulation Efficiency (EE%) and Drug Loading (DL%) of Compound **1**

The encapsulated amount of compound **1** was quantified after freeze-drying and chloroform treatment, which disrupted the lipidic matrix and released the molecule into the organic phase. A spectroscopic estimation of EE% and DL% was carried out by a calibration curve of compound **1** obtained by recording the PL intensity at 350 nm (λ_exc_ 250 nm) of standard chloroform solutions (concentrations ranging from 5 to 0.001 μM). The EE% was calculated according to the formula EE% = (*W*_t_/*W*_i_) × 100, where *W*_t_ is the total amount of compound **1** in the nanoformulation, and W_i_ is the total starting quantity of compound **1** introduced for the preparation. The DL% was calculated as follows: DL % = (*W*_t_/*W*_NLCs_) × 100, where *W*_NLCs_ represents the weight of NLCs.

#### In Vitro Drug Release Study

The in vitro release of compound **1** from NLCs was evaluated using a Transwell permeable support system (48-well plate format). An artificial cellulose acetate membrane (MWC 0.1–0.5 kDa, Fisher Scientific Milano) was inserted into the Transwell insert to separate donor and receptor compartments, allowing diffusion of the released compound **1** while retaining the nanoparticles. Briefly, 200 μL of an NLC dispersion containing compound **1** was placed in the upper chamber, while 400 μL of the release medium was added to the lower chamber. Release studies were performed in a buffer solution at two different pH values (pH 7.4, phosphate buffer saline (PBS); pH 5.0, MES). The system was incubated at 37 °C under orbital shaking at 80 rpm. At predetermined time intervals, the release medium from the lower chamber was collected for spectroscopic analysis and subsequently returned to the same well. Each experiment was performed in triplicate. The cumulative release of compound **1** was determined at each time point and expressed as the cumulative release percentage as a function of time.

#### In Vitro MAO B Inhibition Assay

The inhibition of human recombinant MAO B was measured by means of a fluorescence kynuramine-based protocol.^17^ Experiments were performed in triplicate in black, flat-bottomed polystyrene 96-well microtiter plates (Greiner Bio-One, Kremsmünster, Austria). Briefly, an aliquot of dimethyl sulfoxide (DMSO) or an aqueous solution of the sample under investigation (compound **1**, empty NLCs, **1**/NLCs) was added to the aqueous stock solution of kynuramine and diluted with potassium phosphate buffer. Safinamide was included as a reference inhibitor. Plates were incubated at 37 °C for 20 min (or 6 h) before adding the MAO B solution. After 30 min of incubation at 37 °C, aq. NaOH (2N) was added, and the formation of 4-hydroxyquinoline was quantified with an Infinite M1000 Pro fluorescence multiplate reader (Tecan, Cernusco sul Naviglio, Italy) at excitation/emission wavelengths of 310/400 nm (20 nm slit width for excitation, 30 nm slit width for emission). Inhibitory activities (IC_50_s) were determined by means of nonlinear regressions performed with GraphPad Prism 6.0 software. Results are the mean of at least two independent experiments.

#### MTT Assay

Brain-immortalized endothelial cell line bEnd-3, DI TNC1 astrocytes, and SH-SY5Y human neuroblastoma cell lines were maintained in T-75 flasks (75 cm^2^) in DMEM supplemented with 100 U/mL penicillin, 100 μg/mL streptomycin, 10% FBS, at 37 °C, 5% CO_2_. To assess the biocompatibility of nanoformulations, confluent bEnd-3, DI TNC1 and SH-SY5Y, were seeded at a density of 2 × 10^4^ cells/well in a 96-well plate and treated with the empty NLCs at concentrations ranging from 1.8 to 369 μg/mL or with the compound **1** or **1**/NLCs at concentrations 0.5, 1, 5, 10, 20 μM, with or without CDs. After 24 h of incubation, the cell viability was evaluated by the MTT assay, as reported by Latronico et al.,^31^ and cell viability was expressed as a percentage in comparison to control (CTRL), represented by untreated cells.

#### Reactive Oxygen Species Detection

The detection of reactive oxygen species (ROS) was performed by loading confluent SH-SY5Y cells with 10 μM DCFH-DA in phenol red-free DMEM. After incubation at 37 °C for 30 min and 5% CO_2_, cells were treated for 30 min with H_2_O_2_ (100 μM) and (a) free compound **1**, (b) **1**/NLCs at a concentration of 5 μM, and (c) NLCs at a concentration of 92 μg/mL, corresponding to 5 μM 1/NLCs. Cells treated only with DCFH-DA or H_2_O_2_ represented the negative (CTRL) and positive (H_2_O_2_) controls, respectively.

The PL intensity (PLI) was detected by spectrofluorimetric analysis (λ_exc_ = 485 nm; λ_em_ = 525 nm) using a multiplate reader (Cytation 3 Imaging Reader; BioTek, Winooski, VT, United States). The PLI values were normalized to cell viability, and ROS production was expressed as a relative percentage of PLI versus positive control using the following equation: % ROS production = (PLI _sample_/PLI _H2O2_) × 100.

#### Evaluation of the Ability of Compound **1** and **1**-CD/NLCs to Cross the In Vitro BBB Model

The BBB in vitro model was set by coculturing mouse bEnd-3 cells and DITNC1 as reported by La Spada et al.^1^ Briefly, bEnd-3 cells were seeded at a density of 2 × 10^4^ cells/cm^2^ in the apical compartment of 24-well transwell inserts containing a track-etched poly­(ethylene terephthalate) (PET) membrane (0.4 μm pores) pretreated with rat tail collagen type I (500 μg/mL). DITNC1 astrocytes were seeded at a density of 2.5 × 10^4^ cells/cm^2^ on the underside of the insert membrane coated with 0.01% poly-l-lysine (PLL). The formation of the artificial BBB was assessed by measuring trans-endothelial electrical resistance (TEER) with an epithelial voltammeter. Measurements were performed daily starting from day 2 of coculture to monitor barrier maturation. To obtain accurate resistance measurements, the mean TEER value recorded in control (CTRL), represented by the insert without cells, was subtracted from the values measured in inserts containing the coculture system. TEER values were normalized to the membrane surface area and are expressed as Ω/cm^2^. Paracellular permeability of the BBB was assessed on day 5, corresponding to the time point at which the coculture model reached maximum TEER values (168.48 ± 20.71 Ω/cm^2^). Fluorescein isothiocyanate dextran (FITC–D) was employed as a fluorescent tracer.^31^ For this analysis, 1 mL of FITC-D at a concentration of 200 μg/mL was applied into the upper chamber of the inserts. After 3 h of incubation at 37 °C and 5% CO_2_, supernatants were collected from the upper and lower compartments, and apparent permeability (Pa) was estimated by quantifying FITC-D in the samples by SS PL spectroscopy. The amount of FITC-D permeated was determined by a FITC-D calibration curve obtained by acquiring PL spectra (λ_exc_ 490 nm) of FITC-D water solutions at concentrations ranging from 0.0003 to 0.03 mg/mL. The supernatants were lyophilized, treated with 1 mL of water, and centrifuged at 1500*g* for 15 min to remove the DMEM components. The Pa value of FITC-D permeated across the artificial BBB model was calculated by evaluating the permeability through a blank insert without cells (CTRL) and across the insert containing cells (coculture).^32,33^

Following the assessment of a functional and intact BBB at day 5,^31^ the ability of compound **1**, either in its free form or encapsulated in CD/NLCs (**1**-CD/NLCs), to cross the barrier was subsequently evaluated. Specifically, 20 μM compound **1**, free or encapsulated into CD/NLCs (369 μg/mL), was applied into the upper chamber of the inserts. After 2 h of incubation at 37 °C and 5% CO_2_, supernatants were collected from the upper and lower chambers of the transwell and analyzed to assess the endothelial permeability (Pe) of free compound **1** or **1**-CD/NLCs. The controls (CTRL) were represented by inserts without cells treated under the same conditions.^31^

To evaluate that the BBB maintained its integrity after treatment with compound **1** or **1**-CD/NLCs, the supernatants were collected, the inserts were washed, and the Pa of FITC-D was detected as previously described.^32,33^

The amount of compound **1** that effectively crossed the BBB model was determined by the PL spectra. **1**-CD/NLCs samples recovered from the upper and lower chambers of the transwell were lyophilized and treated with 1 mL of chloroform. The inorganic salts derived from the culture medium were removed by centrifugation (1500*g* for 15 min). Subsequently, the supernatants were collected and the amount of compound **1** was determined by the calibration curve (λ_exc_ 250 nm). The amount of compound **1**, recovered in the lower chamber of the transwell after transmigration through the artificial BBB, was calculated as a percentage with respect to its initial amount detected in the upper chamber of the transwell.

The Pe of **1**-CD/NLCs was calculated by starting from the CDs’ PL spectra. The samples recovered from the upper and lower chambers of the transwell were lyophilized, then treated with 1 mL of chloroform, and centrifuged as above-mentioned. Subsequently, the supernatants were collected and analyzed by PL measurements (λ_exc_ 375 nm). A calibration curve was used to estimate the CD concentration in the samples. The Pe value of **1**-CD/NLCs across the artificial BBB model was calculated by evaluating the permeability through blank insert without cells and the permeability across the insert containing cells, as reported in the literature.^32^

### Statistical Analysis

All statistical analyses were carried out with GraphPad Prism 10.0 (GraphPad Software Inc.) for Windows (Systat Software, San Jose, USA). Outlier analysis was conducted prior to statistical comparisons for all data sets. Outlier detection was performed using the ROUT methods. A *Q* value of 1% was applied to ensure the stringent control of the false discovery rate. No outliers were identified by the ROUT test (*Q* = 1%), and therefore, no data points were excluded from the analysis. Data distribution normality was assessed using the Shapiro–Wilk test prior to applying parametric analyses.

The one-way ANOVA was applied, followed by the Tukey post hoc test to compare multiple experimental groups. For paired comparisons, Student’s *t*-test was used for normally distributed data.

The variable “*n*” represents the number of independent experiments performed using different cell cultures. Within each independent experiment, each data point represents the mean of triplicate technical measurements. Where appropriate, original data were converted into percentage (%) values, as described in the corresponding methods sections. Statistical significance was set at *p* < 0.05. The levels of significance are indicated as follows: ***p < 0.001; **p < 0.01; *p < 0.05. Data are presented as mean ± standard deviation (SD).

## Results and Discussion

Polyethylene glycol (PEG)-functionalized NLCs were developed for compound **1** to enhance aqueous dispersibility and potential brain delivery, improving bioavailability and CNS-targeting while preserving intrinsic pharmacological activity. A series of in vitro investigations were conducted to assess the MAO B inhibition activity of compound **1** following NLC encapsulation as well as the effects of the NLCs on cell viability, antioxidant activity, and translocation efficiency across an artificial BBB model. To enable real-time monitoring of nanocarrier permeation, oil-dispersible CDs were synthesized and coencapsulated with compound **1**. These fluorescent nanoprobes, with intense visible emission and excellent biocompatibility, served as stable optical tracers, enabling in vitro tracking of NLC translocation across the BBB model and providing insights into their brain-targeting potential (Figure 1).^34^

**Figure 1:**
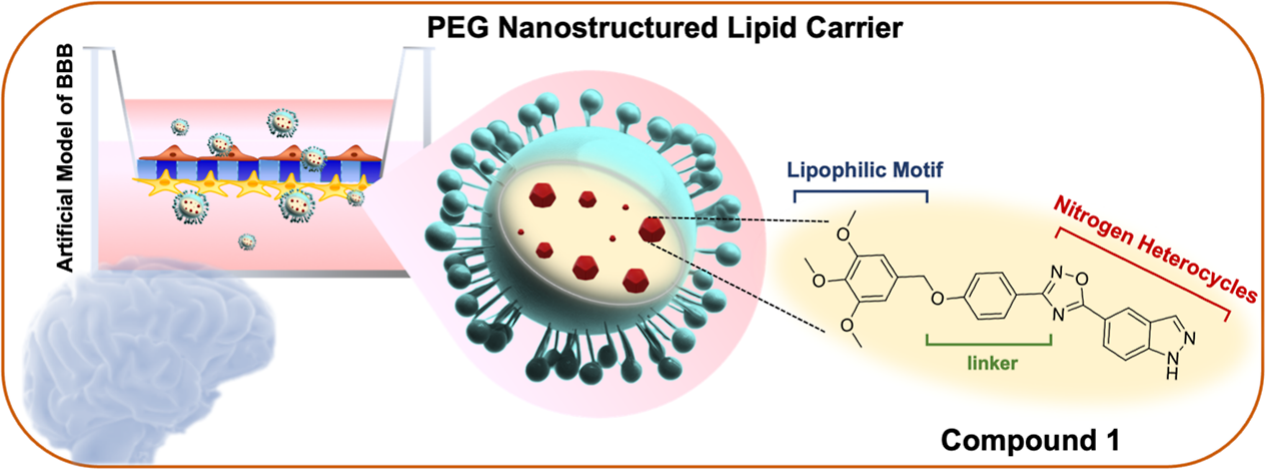
Sketch of NLC-functionalized with PEG and loaded with compound **1**, potentially useful against NDs.

### Compound **1**: From Design to Synthesis

Nitrogen-containing heterocycles have been commonly used as molecular scaffolds to develop MAO inhibitors. In this context, some coauthors have recently published a series of neuroprotective agents based on the 1*H*-indazole nucleus as a privileged motif.^17^ The heterocycle was decorated at C5 by connecting a terminal lipophilic moiety (which could fit the aromatic cavity of MAO B) with different linkers. The highest inhibitory and neuroprotective potencies were achieved by using a terminal 3,4,5-trimethoxyphenyl (TMP) ring linked to a flexible linker. The TMP moiety was inspired by naturally occurring polymethoxylated antioxidants, e.g., tangeretin and quercetin ethers. In particular, the bioisosteric replacement of an amide group at C5 with 1,2,4-oxadiazoles led to the discovery of compound **1** as a potent, reversible, and tight-binding MAO B inhibitor (IC_50_ = 52 nM). Its low activity toward MAO A (14% inhibition at 10 μM concentration, IC_50_ > 10 μM) led to an outstanding selectivity profile showing SI_MAO B_ > 192 (SI = Selectivity Index toward MAO B = IC_50_ MAO A/IC_50_ MAO B). Apart from its promising in vitro inhibitory profile, the physicochemical characterization of compound **1** revealed poor aqueous solubility at physiological pH (S_7.4_ = 17 μM) and high lipophilicity (cLog P = 5.01). Notably, hydrolytic instability was observed only under strongly acidic conditions (pH 2), employed to simulate the gastric environment, whereas the compound remained stable at physiological pH, supporting its suitability for future in vivo administration via a systemic route. The reduced half-life detected at pH 2, compared with biphenyl analogues lacking the –OCH_2_– bridge, is mainly due to the presence of the trimethoxybenzyl ether fragment.^17^ While the acidic instability is confined to gastric-like conditions and does not represent a limitation for systemic use, the extremely low aqueous solubility at physiological pH constitutes a major barrier to further development, severely limiting bioavailability and preclinical future progression of this molecule toward in vivo studies.^17^ Importantly, structure–activity relationships reported in the original study clearly indicated that the trimethoxybenzyl ether moiety is a key pharmacophore element for MAO B inhibition. Indazoles bearing an inverted linker (–CH_2_O– vs –OCH_2_–) or bearing a more rigid biphenyl group (without an oxygen-containing linker) were less active MAO B inhibitors than compound **1**. On this basis, the (nano) formulation approach was envisaged as a tool to bypass the drug-like limitations of compound **1**, increase bioavailability, and improve CNS delivery while preserving the structural features essential for potent MAO B inhibition.

The compound **1** was thoroughly characterized using a combination of complementary optical techniques, namely, UV–vis absorption spectroscopy, PL, and ATR FT-IR spectroscopy (Figure 2).

**Figure 2:**
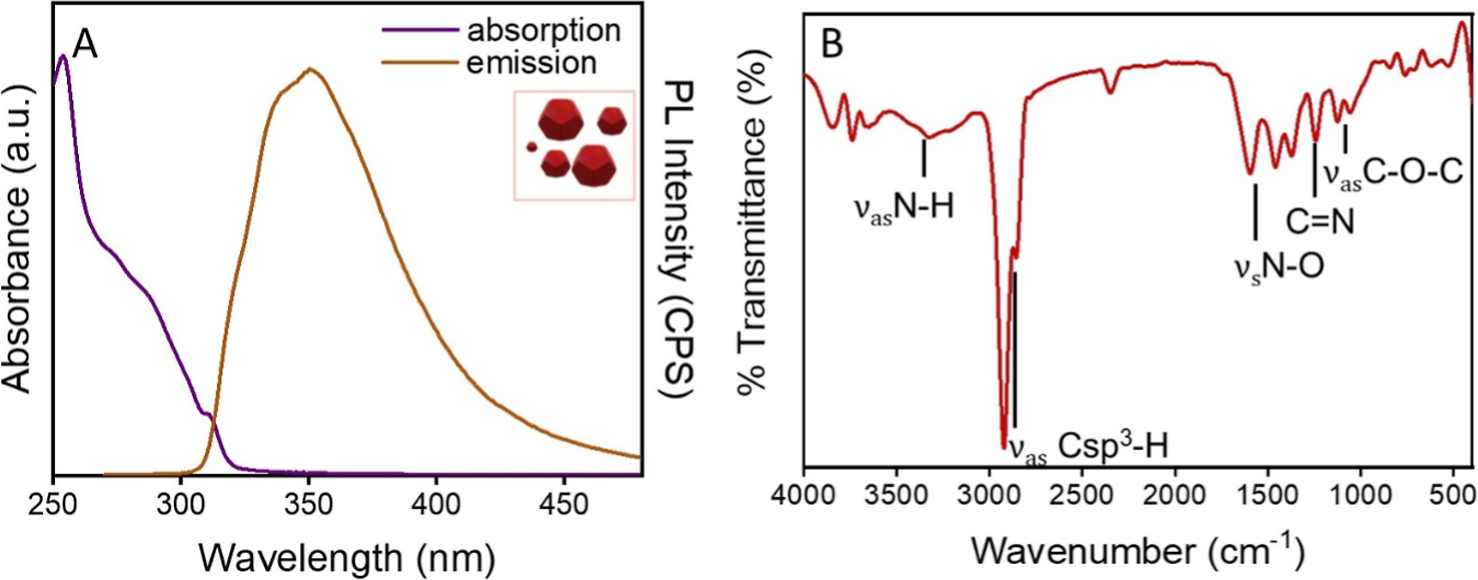
(A) Absorption (purple line) and PL emission (orange line) spectrum (λ_exc_ = 250 nm) and (B) ATR FT-IR spectrum of a chloroform solution of compound **1**.

The UV–vis spectrum of compound **1** in chloroform solution (Figure 2A) displays an absorption band that peaks nearly at 250 nm, accompanied by a shoulder. These features are attributed to π → π* electronic transitions associated with the aromatic moieties of benzene, 1,2,4-oxadiazoles, and 1*H*-indazole units. Upon excitation at 250 nm, the molecule exhibits a broad PL emission band from 300 to 450 nm, with a maximum at approximately 350 nm, consistent with emission from the benzene and 1*H*-indazole fragments within the structure of compound **1**.^35,36^ The ATR FT-IR spectrum of the molecule exhibits characteristic vibrational bands, including the N–H stretching band at ∼3500 cm^–1^ and the aliphatic Csp^3^–H stretching bands at ∼2970 and 2850 cm^–1^. Additionally, signals are observed in the overtone region along with peaks corresponding to the N–O stretching at ∼1600 cm^–1^, the C=N stretching at ∼1250 cm^–1^, and the C–O–C asymmetric stretching at ∼1100 cm^–1^.

### Preparation of NLC-Based Formulations

Three different samples of PEG-stabilized NLCs, encapsulating the compound **1** (**1**/NLCs) (**1**/NLCs 1, **1**/NLCs 2, and **1**/NLCs 3) were prepared at increasing initial concentrations of compound **1** (1, 2, and 3 mg/mL) to investigate the most suited experimental conditions, aiming at optimizing the loading process. After the freeze-drying step, the amount of compound **1** loaded into NLCs was determined by PL spectroscopy, and the DL% and EE% values were calculated (Figure 3A,B). An increase in DL% and a corresponding decrease in EE% are achieved by increasing the concentration of compound **1** from 1 to 3 mg/mL. Indeed, starting from the 2 mg/mL initial solution of the molecule, it is possible to optimize its loading while preserving the EE% of the process. By the calibration curve, the actual concentration of the compound **1** in the nanoformulation **1**/NLCs 2 was determined to be (288 ± 2) μM. Hereafter, the optimized formulation 1/NLCs 2 is referred to as 1/NLC for simplicity and used in subsequent investigations.

**Figure 3:**
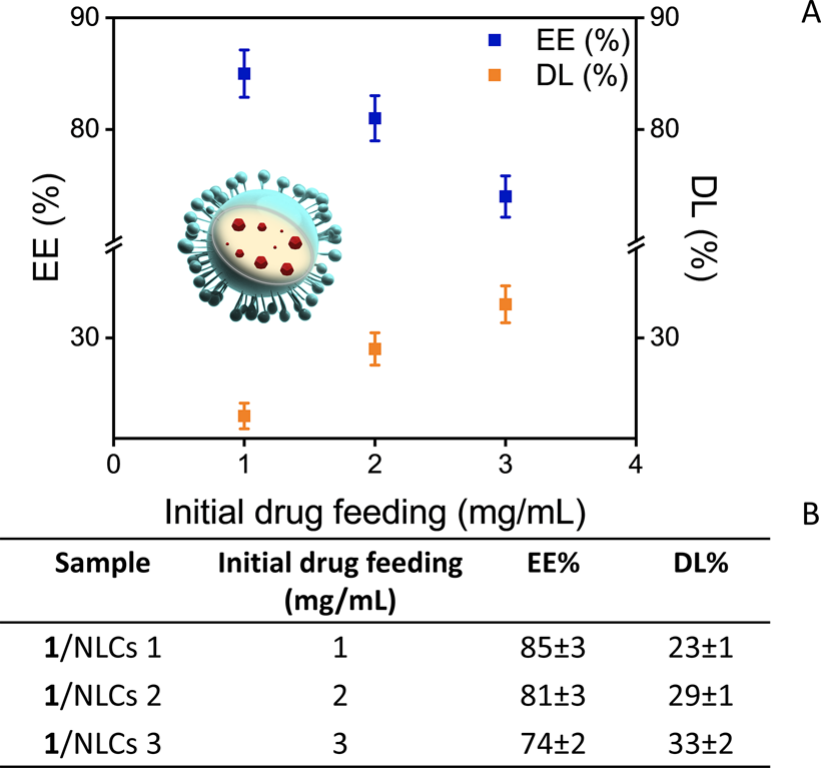
(A) Encapsulation efficiency (EE%) and drug loading (DL%) percentages of compound **1** as a function of the initial drug feeding (mg/mL), based on quantitative analysis of PL spectra. (B) EE% and DL% values of compound **1** determined across different **1**/NLC formulations. All reported data are presented as mean values ± SD (three replicates).

The optimized formulation **1**/NLCs was systematically characterized in terms of particle size, colloidal stability, and morphology using DLS, ζ-potential measurements, and TEM analysis, and compared with the empty counterpart prepared using the same experimental procedure but without adding compound **1** (Figure 4).

**Figure 4:**
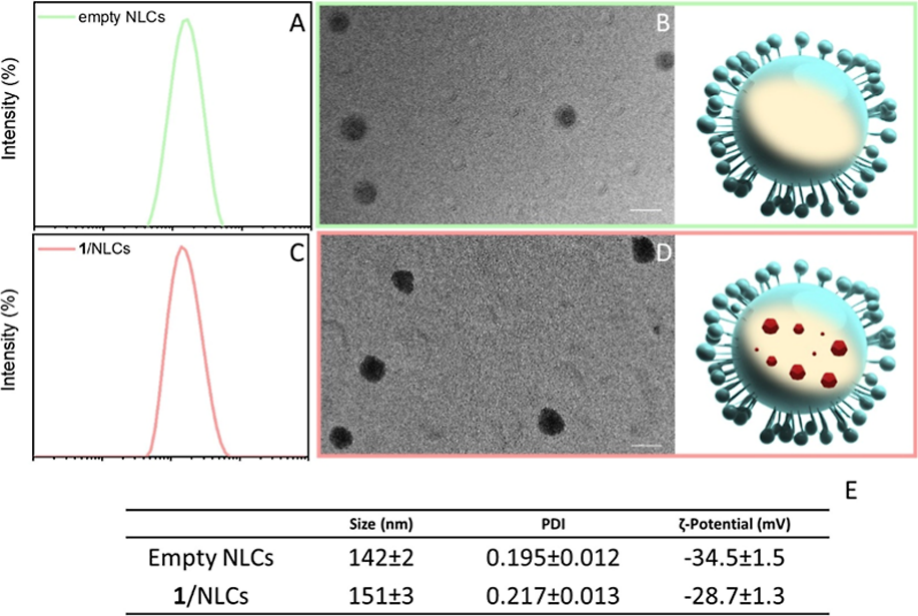
Size distributions by intensity, performed by DLS measurements, of (A) empty NLCs and (B) **1**/NLCs. Representative TEM micrographs (scale bars 100 nm) and respective schematic sketch of (B) empty NLCs and (D) **1**/NLCs. Drawings are not to scale. (E) Summary of the size (average hydrodynamic diameter), PDI, and ζ-potential values of empty NLCs and **1**/NLCs. All reported data are presented as mean values ± SD (three replicates).

DLS analysis showed that the **1**/NLCs exhibit a mean hydrodynamic diameter of (151 ± 3) nm, while empty NLCs display a slightly smaller size of (142 ± 2) nm, both characterized by monomodal size distributions (Figure 4A,C,E). The PDI values (∼0.20) indicate a narrow and homogeneous size distribution typical of well-formed lipid nanocarriers. These parameters were reproducible across independent batches, and DLS intensity distribution profiles consistently revealed a single well-defined population without evidence of secondary peaks or aggregation phenomena. TEM micrographs confirmed the presence of well-defined, round-shaped nanoobjects with sizes consistent with the DLS results (Figure 4B,D). ζ-Potential measurements indicated a negatively charged surface for the empty NLCs, which is likely attributable to the anionic components of the lipid matrix used for NLC preparation. Upon encapsulation of compound **1**, a slight shift toward more positive ζ-potential values was observed, suggesting that a very small fraction of compound **1** may be associated with the NLC surface (Figure 4E).

Stability studies conducted on **1**/NLCs stored at 4 °C for three years showed no significant changes in particle size distribution or PDI, as determined by DLS, indicating good physical stability under these storage conditions (see Supporting Information, Figure S2). The ζ-potential values also remained essentially unchanged over time, and the dispersions appeared visually clear, with no color changes or detectable signs of aggregation or phase separation during storage (see Supporting Information, Figure S1B). These results are consistent with the structural features of NLCs, in which the partially crystalline and structurally disordered lipid matrix contributes to colloidal stability and prevents particle coalescence or drug expulsion during storage.^37^

Release studies of compound **1** from **1**/NLCs were conducted under physiological (pH 7.4) and endosomal-like (pH 5.0) conditions, which are directly relevant to the envisaged future administration route (systemic administration) and intracellular trafficking following NLC uptake (Figure 5A). The release profiles showed a sustained and incomplete release over 72 h. At pH 5, a cumulative release of approximately 40% was observed, compared to 28% at pH 7.4. Both media exhibited a limited initial burst (<6%), followed by a slower, diffusion-controlled release reaching a plateau, indicative of strong compound **1**-lipid interactions and depot-like behavior. The incomplete release in both media is consistent with the lipophilic nature of compound **1** and its high affinity for the solid lipid matrix. The higher release at pH 5 likely reflects enhanced partitioning of compound **1** into the aqueous phase under mildly acidic conditions. Overall, the profiles indicated high formulation stability under physiologically similar buffer conditions and confirmed that **1**/NLCs provide sustained release while retaining a fraction of compound **1** within the lipid core. Based on literature reports, complete release is expected in the presence of human serum, likely due to protein interactions and enzymatic destabilization of the lipid matrix.^32^

**Figure 5:**
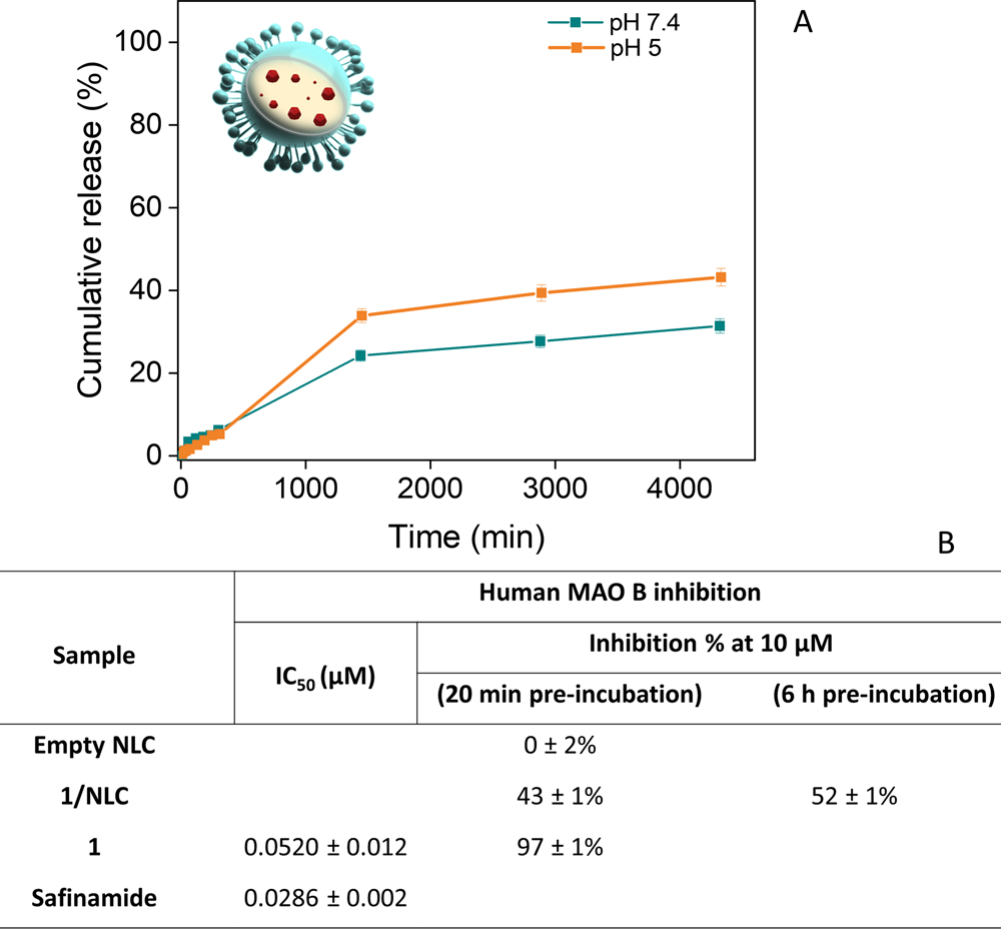
(A) In vitro release profiles of compound **1** from **1**/NLCs at 37 °C at pH 7.4 (dark cyan line) and pH 5 (orange line). (B) IC_50_ values for human recombinant MAO B inhibition for compound **1**, empty NLCs, and **1**/NLCs after different preincubation times (20 min and 6 h) prior to enzyme addition. Compound **1** was tested at 10 μM, and safinamide was used as a reference inhibitor. Experiments were performed in triplicate. Values are expressed as mean ± SD, three replicates.

To assess the pharmacological activity of compound **1** following NLC encapsulation, in vitro MAO B inhibition was evaluated using the kynuramine-based assay previously applied for screening of compound **1**.^17^ Free compound **1**, **1**/NLC (at equivalent concentration of compound **1**), and empty NLCs were compared, with safinamide as a reference (Figure 5B). Empty NLCs showed no inhibition (0 ± 2%), confirming that the carrier does not interfere with the assay. Free compound **1** exhibited potent inhibition (IC_50_ = 0.052 ± 0.012 μM; 97 ± 1% at 10 μM), consistent with prior reports. In contrast, intact **1**/NLC showed limited inhibition (43 ± 1% at 10 μM, 20 min of preincubation), increasing only modestly after 6 h of preincubation (52 ± 1%), in line with independent release studies indicating <5% release of compound **1** under protein-free conditions. This activity is attributed to the initial burst release of surface-associated compound **1**. These results demonstrated that NLC encapsulation does not chemically inactivate compound **1**; rather, the formulation’s controlled-release properties limit the immediate availability of compound **1** in short-term, protein-free assays. The NLC system is intended to enhance the solubility, stability, and delivery of compound **1** under biologically relevant conditions, not to act as a direct MAO B inhibitor. Short-term enzymatic assays do not capture the dynamic release expected in biological fluids, where protein interactions facilitate the release of compound **1**. Thus, the modest inhibition observed reflects the slow, controlled-release behavior of the NLC, consistent with its role as a delivery platform.

Although **1**/NLC is the formulation intended for future therapeutic applications, an optically traceable nanosystem was developed by coloading luminescent CDs and compound **1** (**1**-CD/NLCs) to allow tracking of NLC transport across the in vitro BBB model. Fluorescence CDs hold distinctive advantages marked by multicolor emissions, tunable optical properties, excellent photostability, and prominent biocompatibility, making them promising scaffolds for bioimaging^38,39^ and real-time tracking of drugs.^40^ To obtain luminescent NLCs, first, oil-dispersible CDs were synthesized by the carbonization of citric acid in the presence of HDA in a high-boiling organic solvent, and their morphological and optical properties were investigated (Figure 6A,B). The TEM image reported in the inset of Figure 6A shows the presence of spheroidal nanostructures of 3.1 nm in size (σ = 25%), as estimated by measuring a statistically relevant number of nano-objects. According to A. Panniello et al.,^30^ the presence of amine-containing passivating agents improves the PL emission of CDs, via the formation of molecular fluorescent derivatives alongside the carbonization process, resulting in a remarkable PL QY. Figure 6A reports the UV–vis absorption spectrum of the synthesized CDs, indicating an intense absorption signal in the UV range (λ < 300 nm), whereas two absorption bands are detected in the visible range at 360 and 450 nm. Such absorption signals can be ascribed to transitions associated with the sp^2^ domains of the carbonaceous core as well as to transitions related to surface functional groups and to molecular fluorophores forming during the synthesis reaction.

**Figure 6:**
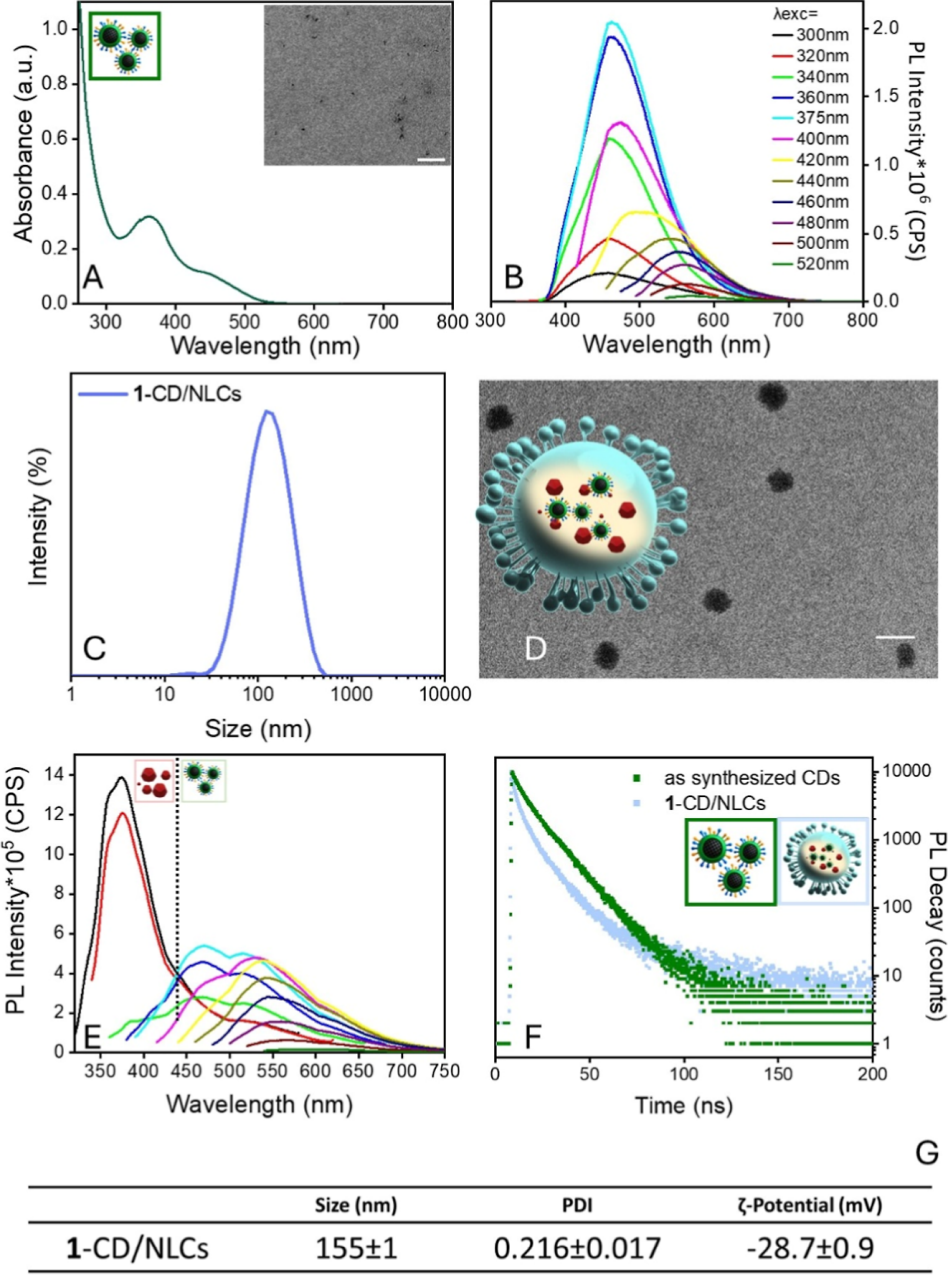
(A) UV–vis and (B) PL emission spectra of a CD dispersion in chloroform. The PL emission spectra are recorded at the various excitation wavelengths as indicated in the legend. In the inset, the TEM micrography of CDs is reported (scale bar 100 nm). (C) Size distributions by intensity, performed by DLS measurements, and (D) representative TEM micrograph (scale bar 100 nm) along with the respective schematic sketch of **1**-CD/NLCs. (E) PL spectra of the **1**-CD/NLC formulation in aqueous solution at various excitation wavelengths. (F) TR-PL spectra measured at λ_exc_ = 375 nm on the emission peak at 460 nm and for as-synthesized CDs and **1**-CD/NLCs dispersed in chloroform and water, respectively. Drawings are not to scale. (G) Summary of the size (average hydrodynamic diameter), PDI, and ζ-potential values of 1-CD/NLCs. All reported data are presented as mean values ± SD (three replicates).

In the PL emission spectra (Figure 6B), depending on the excitation wavelength, the contributions of two fluorescence bands can be observed: the first one, more intense, in the blue region centered at 435 nm, whereas the second one in the yellow region at 575 nm. Specifically, at increasing λ_exc_, a red shift of the PL peak maximum is observed, thus confirming the well-known excitation wavelength-dependent fluorescence of CDs.^30^ As the PL emission depends on the excitation energy, the absolute QY of CDs also slightly varies, reaching maximum values of 38% under a 375 nm excitation wavelength.

The hydrophobic character of the CDs, imparted by the long aliphatic chains of surface-passivating HDA molecules exposed to the surrounding medium, renders these nanostructures inherently oil-soluble (Figure S3, see Supporting Information) and thus suitable as optical tracers into the lipid core of NLCs.

For the preparation of **1**-CD/NLCs, 5 mg of CDs and an initial compound **1** concentration of 2 mg/mL were used, as described in the Materials and Methods section. The sample was lyophilized to determine the amount of effectively encapsulated compound **1**, and quantitative analysis was performed by PL spectroscopy, yielding an average concentration of (70 ± 3) μM. From the PL spectrum (λ_exc_ = 250 nm) and previously described equations, the EE% and DL% were calculated as (57 ± 2)% and (20 ± 3), respectively, showing a decrease compared to the initial formulation (81 ± 3%; 29 ± 1%). This reduction can be attributed to partial occupation of the hydrophobic core by CDs, limiting the solubilization volume available for compound **1**, and reflects a volumetric accommodation limit rather than changes in NCL stability or surface properties.^41–43^ Additionally, to comprehensively characterize the formulation, the amount of effectively embedded CDs was quantified using a calibration curve obtained via PL spectroscopy (Figure S4; see Supporting Information).

DLS analysis and ζ-potential measurements revealed a monomodal size distribution, with an average hydrodynamic diameter of (155 ± 2) nm and a surface charge of (−28.7 ± 9) mV, fully consistent with the values obtained for the formulation without CDs (Figures 4C,E and 6C,G). TEM micrographs also confirmed the formation of well-defined, round-shaped nanoparticles with sizes in good agreement with the DLS results, taking into account the inherent differences between the two techniques (Figure 6D).

The emission spectra recorded at increasing excitation wavelengths (Figure 6E) reveal the presence of both compound **1** and CDs within the formulation. Specifically, at lower excitation wavelengths (300–320 nm), the spectral profiles and emission maxima markedly differ from those of the as-synthesized CDs (Figure 6B), closely resembling the emission characteristics of compound **1** (Figure 2A). Conversely, spectra acquired at higher wavelengths display typical wavelength excitation-dependent PL features associated with CDs; however, both the emission shape and intensity are altered with respect to the pristine nanoparticles (Figure 6B), as a reasonable consequence of the distinct chemical environment within the lipid matrix of the nanocarrier system.

To further elucidate the luminescence properties of **1**-CD/NLCs, TR-PL measurements were conducted on both the as-prepared CDs and the **1**-CD/NLC samples (Figure 6F). The PL decay curves were best fitted by a multiexponential fitting function, yielding average lifetimes (τ_avg_) of (8.9 ± 0.3) ns for the pristine CDs and (4.3 ± 0.3) ns for the **1**-CD/NLCs. The reduction in lifetime observed for CDs embedded in NLCs and dispersed in water, as compared to pristine CDs in chloroform, suggests the presence of nonradiative recombination pathways of the excited electrons. These pathways are likely attributed to changes in the chemical environment and dispersing medium when passing from an organic solvent to a lipid-based aqueous system. Additionally, the decay curve of the embedded CDs does not approach zero, reasonably due to residual scattering effects from the NLC matrix.

### Effect of NLC-Based Formulations on Astrocytes and Endothelial Cell Viability

Prior to evaluating the ability of compound **1**, to cross the BBB, before and after its encapsulation in lipid-based nanocarriers, preliminary studies were carried out to evaluate the biocompatibility of the prepared NLC-based formulations on DI TNC1 and bEnd-3 cells, employed to establish the in vitro BBB model. Both immortalized nontumorigenic astrocytes (DI TNC1) and nonmalignant brain endothelial cells (bEnd-3) show many similarities to primary astrocytes and brain endothelial cells, respectively, and are therefore particularly useful for neurodegenerative disease studies and for the setting up of artificial BBBs. As shown in Figures 7 and 8, the viability of both cell populations treated for 24 h with the free compound **1** was comparable to the control (100%). Treatment with empty NLCs, **1**-CD/NLCs, or **1**/NLCs induced only a moderate reduction in the cell viability of the two cell lines, with values remaining above 60% across the tested concentrations. These findings were further supported by the microscopic evaluation of cell morphology, which revealed no apparent signs of cytotoxic damage (Figures 7 and 8). Both astrocytes (Figure 7) and endothelial cells (Figure 8) exposed to the tested formulations retained typical healthy morphologies with preserved membrane integrity and no evidence of cell shrinkage or detachment. Overall, these results demonstrate that the NLC-based formulations exhibit good biocompatibility and are well tolerated by both cell types under the explored experimental conditions.

**Figure 7:**
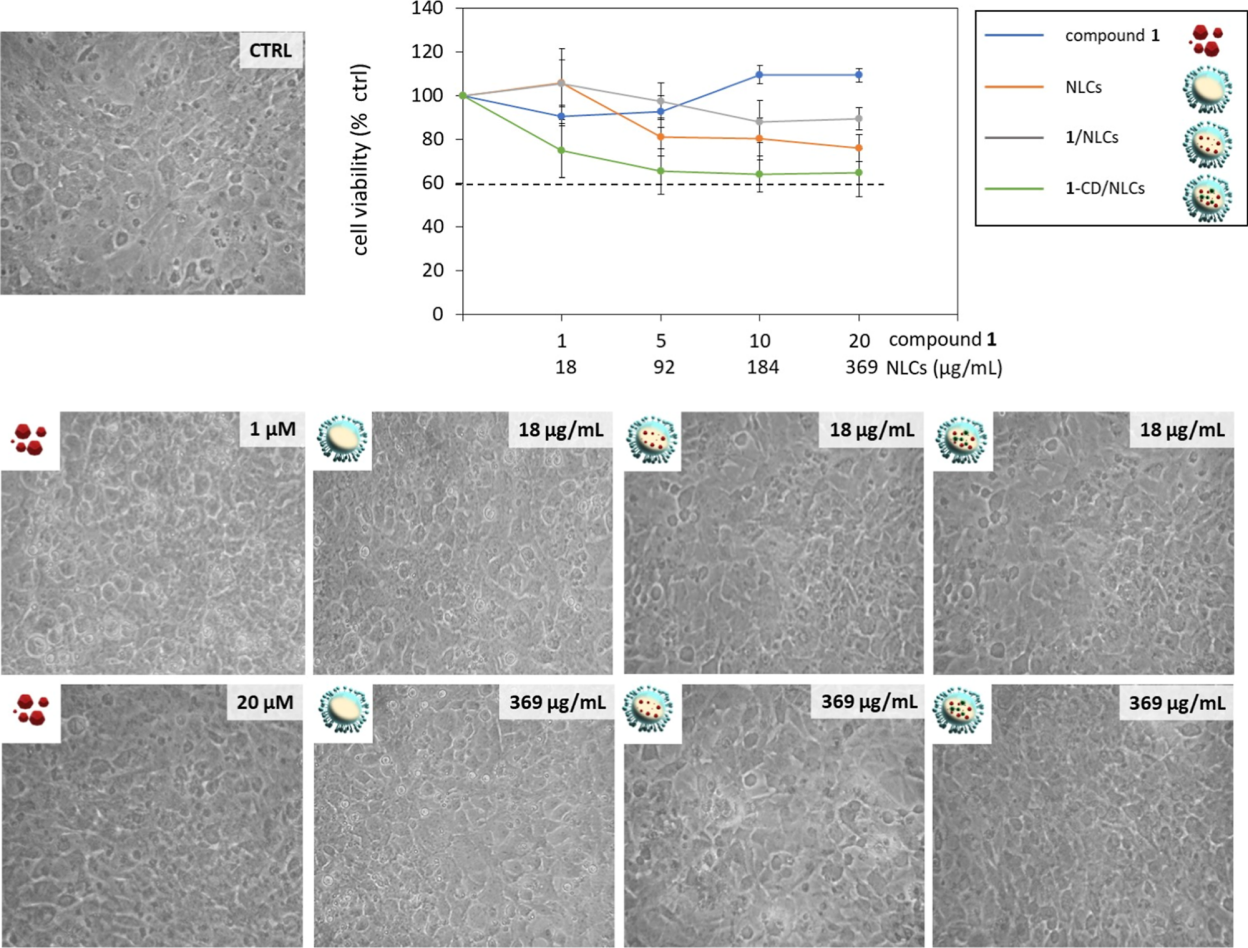
Effect of compound **1**, empty NLCs, **1**/NLCs, and **1**-CD/NLCs on DI TNC1 viability. The representative images show the morphology of the DI TNC1 observed by phase contrast microscopy (20× magnification) after 24 h of treatment with compound **1**, empty NLCs, **1**/NLCs, or **1**-CD/NLCs at the indicated concentrations. The graph reports cell viability, assessed by the MTT assay, expressed as a percentage of surviving cells compared to untreated astrocytes in serum-free DMEM, set as control (CTRL, 100%). The doses of the NLC-based formulations resulting in a cell survival below 60% were considered toxic. Data represent mean ± SD of *n* = 3 independent experiments on different cell populations.

**Figure 8:**
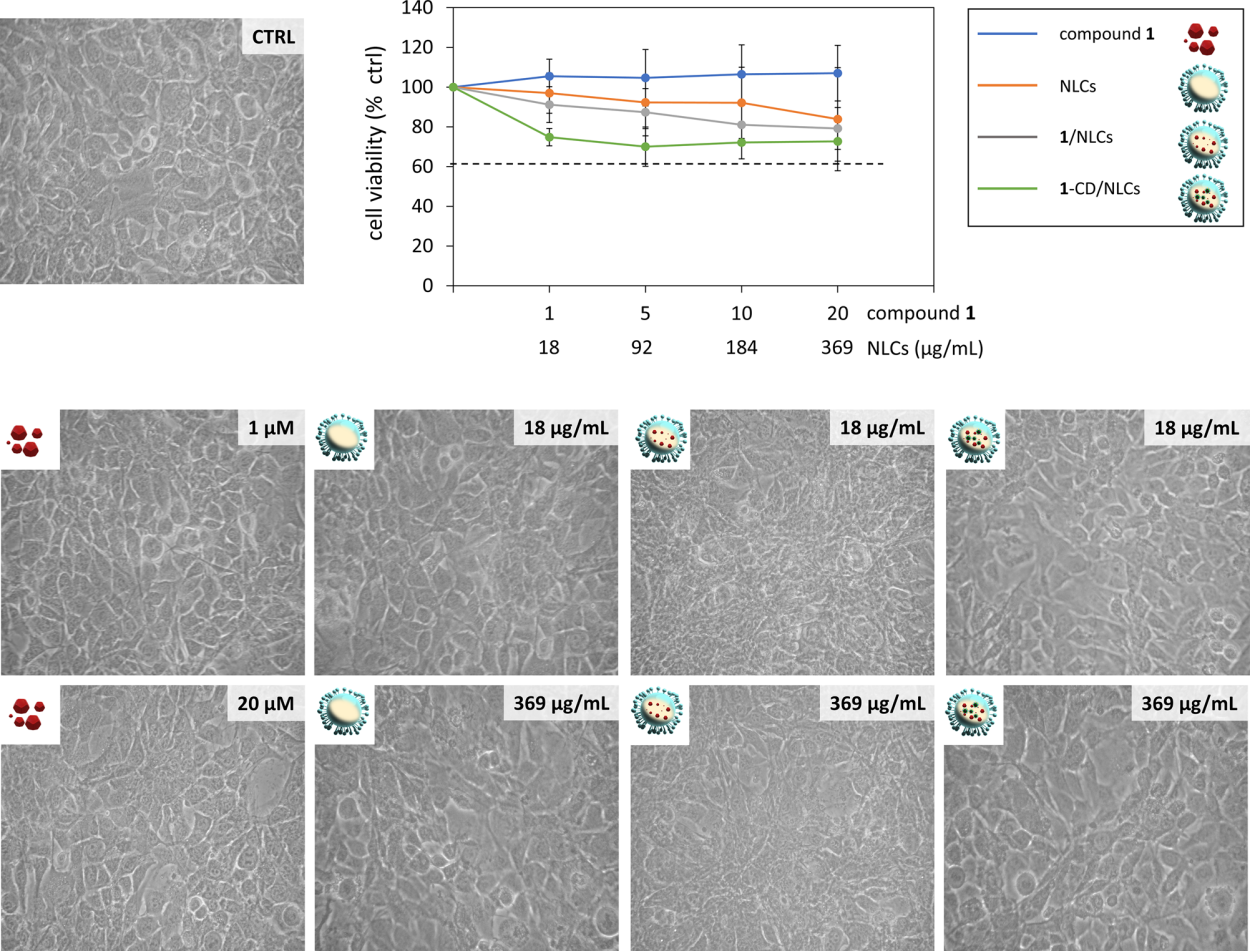
Effect of compound **1**, empty NLCs, **1**/NLCs, and **1**-CD/NLCs on bEnd-3 viability. The representative images show the morphology of the bEnd-3 observed by phase contrast microscopy (20× magnification) after 24 h of treatment with molecule **1**, empty NLCs, **1**/NLCs, or **1**-CD/NLCs at the indicated concentrations. The graph reports cell viability, assessed by the MTT assay, expressed as a percentage of surviving cells compared to untreated astrocytes in serum-free DMEM, set as control (CTRL, 100%). The doses of the NLC-based formulations resulting in a cell survival below 60% were considered toxic. Data represent mean ± SD of *n* = 3 independent experiments on different cell populations.

### Ability of Compound **1** Free or Encapsulated in NLCs (**1**/NLCs) to Counteract the ROS Production in SH-SY5Y Neuroblastoma Cells

In a previous study, Rullo et al.^17^ tested the ability of free compound **1** to counteract the production of ROS in the SH-SY5Y neuroblastoma cell line, one of the most widely used cellular models for studying neurodegenerative diseases.^44,45^ Although compound **1** is an MAO B inhibitor and MAO B is predominantly expressed in astrocytes, to ensure experimental continuity and direct comparability with our previous work,^17^ in this study, the same cell line of SH-SY5Y was employed in ROS assays to assess whether the encapsulation of compound **1** within NLCs affects its antioxidant performance.

Before testing the effect on ROS production, preliminary experiments were carried out to assess the effect of the prepared nanoformulations on the viability of the SH-SY5Y cells. As shown in Figure 9A, the cell viability of SH-SY5Y treated with **1**/NLCs (corresponding concentrations of free compound **1**, ranging from 0.5 to 20 μM) was comparable to that of cells treated with free compound **1**.

**Figure 9:**
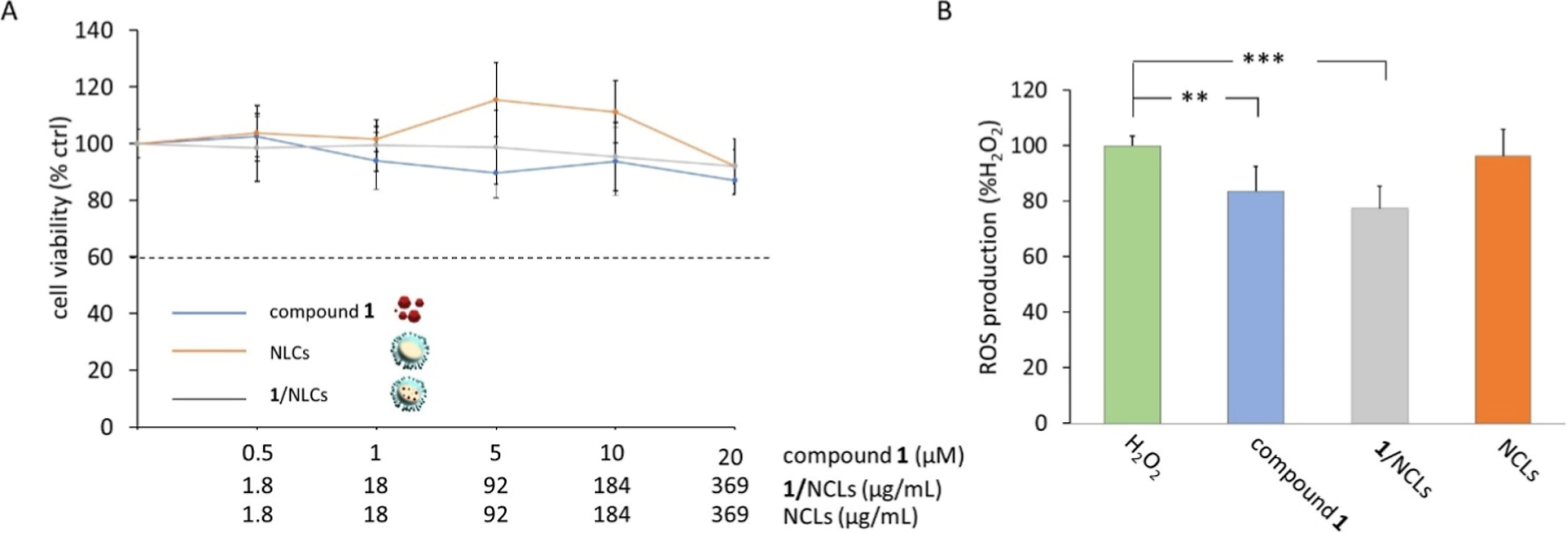
(A) Cell viability, assessed by the MTT test, of SH-SY5Y treated for 24 h with compound **1** free or encapsulated in NLCs (**1**/NLCs) or NLCs at the indicated concentrations. The results are expressed as a percentage of surviving cells compared to untreated cells in serum-free DMEM (CTRL, 100%). The doses of the samples resulting in a cell survival below 60% were considered toxic. (B) Levels of reactive oxygen species (ROS) were assayed by measuring the changes of the fluorescent signal of 2′,7′-dichlorofluorescein (DCFA) as reported in the Materials and Methods section. SH-SY5Y cells were treated with 100 μM H_2_O_2_ in the presence of 5 μM free compound **1** or **1**/NLCs or with 92 μg/mL NLCs (corresponding to 5 μM 1/NLCs). The positive control (H_2_O_2_) was represented by SH-SY5Y cells treated with H_2_O_2_ alone. The ROS production was expressed as a percentage (%) of photoluminescence (PL) intensity in comparison to the positive control. The values represent mean ± SD of *n* = 3 experiments performed on different cell populations; asterisks indicate values statistically significantly different in comparison to H_2_O_2_ (one-way ANOVA followed by Tukey’s post hoc test; **p < 0.01, ***p < 0.001).

To test the antioxidant activity, the cells were treated with compound **1**, both free and encapsulated in NLCs (**1**/NLCs), at the same concentration (5 μM) used by Rullo and colleagues,^17^ which was previously shown to exert significant antioxidant activity without inducing cytotoxic effects. As shown in Figure 9B, compound **1** significantly reduced ROS levels compared with those of H_2_O_2_-treated cells (*p* = 0.0014), confirming its intrinsic antioxidant activity. Similarly, compound 1 encapsulated in NLCs maintains its ability to inhibit ROS production (*p* = 0.0001). Moreover, the absence of a significant reduction in ROS levels in cells treated with empty NLCs compared to H_2_O_2_-treated cells (*p* = 0.1917) suggests that the antioxidant effect observed with **1**/NLCs is attributable to compound **1** itself rather than to the lipid nanocarrier components, as further supported by the significant difference observed between empty NLCs and **1**/NLC (*p* = 0.0049). The results highlight that encapsulating compound **1** in NLCs enhances its ability to inhibit ROS production (Figure 9B). The antioxidant activity likely reflects the combined contribution of the TMP moiety, intrinsic redox properties of the indazole scaffold, reduced H_2_O_2_ generation upon MAO-B inhibition, and direct radical-scavenging activity. The 10% increase of inhibition of ROS production exerted by **1**/NLCs compared with free compound **1** could be attributed to the ability of the NLCs to improve the intracellular accumulation of compound **1** by enhancing cell membrane permeation or by modulating aqueous solubility. In this respect, Mihailova and colleagues^46^ demonstrated that NLC uptake by SH-SY5Y may follow the transcytosis pathway of internalization due to the hydrophobic nature of the lipid carriers, making them suitable candidates for facilitated transport through apical cell membranes. In addition, Shubra et al.^47^ reported that poloxamers (such as Kolliphor P188) could be incorporated into cell membranes, modifying their microviscosity. Moreover, they can decrease ATP levels, thus depleting the activity of efflux transporters, which could result in enhanced BBB penetration. These results are in accordance with the above-mentioned investigations, suggesting that, most likely, poloxamer contributes to cellular uptake via endocytosis, rather than passive diffusion.^48^

### Ability of Compound **1** Free and Encapsulated in NLCs (**1**-CD/NLCs) to Cross the Artificial Model of the BBB

Finally, an in vitro BBB model was employed to evaluate the ability of the lipid-based nanoformulation to enhance the permeability of the neuroprotective compound **1** across the BBB. The model was established by coculturing murine bEnd-3 cells with DI TNC1, following the protocol previously described in Latronico et al.^31^ (Figure 10A). This BBB model was intentionally selected to ensure methodological consistency with our previously established and validated in vitro BBB used to investigate the permeability and translocation of other synthetic compounds acting as MAO B inhibitors.^1^ This consistency is particularly relevant in the context of nanoparticle-mediated delivery systems, where experimental reproducibility represents a critical parameter. Although the model used in this study may underestimate the physiological tightness of the BBB, as reflected by lower TEER values and the lack of full structural and functional complexity including the contribution of pericytes, basement membrane organization, and shear stress associated with blood flow, it provides a consistent platform to reliably compare the transendothelial transport and nanocarrier-mediated delivery efficacy.

**Figure 10:**
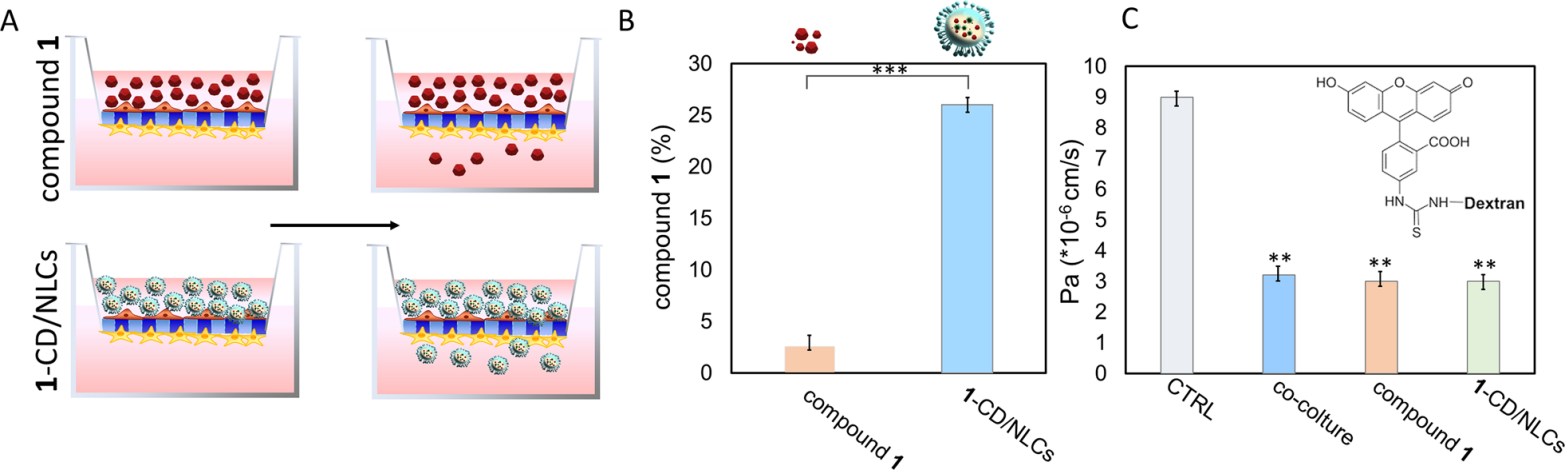
(A) Sketch of the in vitro experiments performed to investigate the penetration of free compound **1** and **1**-CD/NLCs through the artificial BBB. (B) Histograms representing the amounts of free compound **1** and **1**-CD/NLCs through the BBB model, calculated as a percentage of the content of compound **1** in the lower chamber with respect to its initial amount in the upper chamber. Statistical differences were assessed by Student’s *t*-test (*** = *p* < 0.001) (C) Histograms representing the average values of the Pa coefficient of (FITC-D), relative to CTRL, represented by inserts without cells and by inserts containing bEnd3/astrocyte coculture, assessed before and after the passage of free compound **1** or **1**-CD/NLCs. The Pa coefficients were calculated as the ratio between the amount of FITC-D passed in the lower chambers and that remaining in the upper chambers of the transwells. Statistical differences were assessed by one-way ANOVA followed by Tukey’s post hoc test. Asterisks indicate values statistically significantly different in comparison to CTRL (** = *p* < 0.01). Data represent mean ± SD of *n* = 3 independent experiments on different cell populations.

For BBB permeability experiments, to ensure adequate analytical sensitivity for quantification after translocation across the barrier, 20 μM compound **1**, either in its free form or encapsulated within CD/NLCs (369 μg/mL), namely, **1**-CD/NLCs, was added to the apical compartment of the transwell inserts. Notably, no significant differences were observed in the hydrodynamic diameter, PDI, or ζ-potential between **1**/NLCs and **1**-CD/NLCs (Figures 4C,E and 6C,G). The unchanged surface charge suggests that CDs are predominantly confined within the lipid core rather than exposed at the NLC interface. Since nanoparticle–BBB interactions and cellular uptake are largely governed by particle size, surface charge, and colloidal stability, the two formulations are expected to display comparable behavior in terms of barrier interaction. After 2 h of incubation, the amount of compound **1** and **1**-CD/NLCs crossing the cell monolayer was quantified by PL spectroscopy. The 2 h incubation time for the BBB permeation experiments was selected based on preliminary time-course studies identifying this time point as the optimal condition to preserve cell viability and barrier integrity while allowing reliable detection of differences in translocation between free compound **1** and **1**-CD/NLC-encapsulated compound **1**. As shown in Figure 10B, a significantly higher fraction of compound **1** crosses the BBB when delivered via NLCs (26 ± 2%) compared with the free molecule (2.6 ± 1.1%) (*p* < 0.001), underscoring the enhanced permeation efficiency of the nanoformulations. The Pe coefficient of the **1**-CD/NLC formulation determined by PL signals of the encapsulated CDs was (6.7 ± 0.5) × 10^–6^ cm s^–1^, confirming the effective translocation of the nanoformulations across the barrier. Notably, the amount of compound **1** encapsulated in the NLCs detected in the lower compartment (26 ± 2%) corresponds to approximately 5 μM, which is comparable to the concentration used in the ROS experiments.

To ensure that the crossing of the nanoformulations did not compromise the integrity of the cell-based layer, the Pa of fluorescein isothiocyanate–dextran (FITC-D, 4 kDa), a marker of paracellular transport, was evaluated in the model both prior to and following treatment. As shown in Figure 10C, the Pa coefficient calculated for the coculture BBB model following administration of either free compound **1** or the **1**-CD/NLC formulation remained statistically unchanged at (3.0 ± 0.4) × 10^–6^ cm/s and (2.9 ± 0.2) × 10^–6^ cm/s, respectively, in comparison to the Pa value prior to treatment (3.2 ± 0.3) × 10^–6^ cm/s. This value was significantly lower (*p* < 0.01) than that measured for control inserts coated with poly-l-lysine (PLL) and collagen type I in the absence of cells [(9.0 ± 0.2) × 10^–6^ cm/s]. These results collectively confirm that the NLC-based delivery system enables enhanced translocation of the pharmacologically active molecule across the BBB compared to the free compound without compromising the structural or functional integrity of the in vitro BBB model. In a recent paper, other authors demonstrated that the encapsulation in solid lipid nanoparticles of Donepezil, a cholinesterase inhibitor used in the treatment of AD, improved its permeation, pharmaceutical efficacy, and stability, confirming the usefulness of this approach in targeting potential new synthetic drugs at the CNS level overcoming the difficulty of crossing the BBB.^49–51^ A comparison with previously reported studies indicates that lipid-based formulations developed for drugs such as donepezil and rivastigmine generally achieve enhancement ratios in the range of approximately 2–5-fold.^52,53^ Within this context, the ∼10-fold increase observed in our study suggests a potentially improved performance of the present system in enhancing drug transport across the barrier. This effect may be associated with several formulation parameters, including the optimized particle size (∼150 nm), the selected lipid composition of the NLCs, the lipophilic nature of compound **1**, and the presence of Kolliphor P188, which may contribute to nanostructure stabilization and potentially modulate efflux processes.

These outcomes are particularly significant considering that less than 2% of all FDA-approved small-molecule drugs are able to cross an intact BBB, despite the fact that such compounds are often rationally designed by balancing lipophilicity and molecular weight to improve their likelihood of CNS penetration.^54^ Indeed, Lipinski et al.^55^ identified molecular weight and lipophilicity as critical physicochemical parameters governing the passive diffusion of small molecules across the biomembrane. CNS-active compounds generally exhibit substantially lower molecular weight cutoffs compared to other systemic therapeutics. In particular, BBB permeability has been shown to decrease by approximately 100-fold when the molecular weight increases from 200 to 450 Da, representing a key limitation in the design of drugs intended for brain delivery. Nevertheless, to achieve optimal therapeutic efficacy, small molecules must exhibit not only high potency and selectivity for their molecular target but also sufficient bioavailability at the target site.^54^

A further challenge lies in the nonspecific binding of small molecules to serum proteins, notably human serum albumin, which can increase the effective molecular size and hinder BBB permeation.^56^ This reversible binding reduces the free drug fraction available for diffusion across the BBB, as only unbound molecules can traverse the barrier.^57,58^ Additionally, many intravenously administered small molecules are subjected to rapid metabolic degradation by serum enzymes, which further diminishes their availability to the brain.^59^

Moreover, the high expression of P-glycoprotein (P-gp), a multidrug-resistance transporter located at the luminal surface of the BBB endothelium, constitutes a major obstacle to CNS drug delivery. Acting as an ATP-dependent efflux pump, P-gp actively exports a wide array of xenobiotics from the brain back into the bloodstream, thereby limiting CNS accumulation and contributing to neuroprotection.^60^

Our findings demonstrate that the encapsulation of compound **1** into Kolliphor P188-stabilized NLCs improves its BBB permeability and pharmacological availability, thereby overcoming several of the aforementioned limitations. Although the precise mechanisms underlying this enhancement were not specifically investigated in the present study, it is conceivable that they may involve modulation of efflux transporters and/or facilitation of endogenous transport pathways. Notably, the surfactant Kolliphor P188 has been shown to inhibit P-gp function and enhance BBB translocation in other experimental settings.^61–65^ However, whether compound **1** is a P-gp substrate remains to be elucidated.

It should be noted that all findings reported herein are derived exclusively from in vitro experimental models. Future studies will therefore need to evaluate the systemic administration potential of this nanoformulation through stability and biocompatibility assessments under physiologically relevant conditions, including studies in whole blood and serum. In addition, comprehensive in vivo pharmacokinetic investigations, such as plasma exposure profiling, brain biodistribution, and evaluation of BBB penetration efficiency, will be required. Validation of therapeutic efficacy and safety in appropriate animal models of neuroinflammation and NDs will also be necessary. As no in vivo studies have been performed, conclusions regarding the therapeutic applicability of the present system remain preliminary. Nevertheless, these results provide a proof-of-concept framework supporting further preclinical development of this nanoformulation as a potential CNS delivery strategy.

## Conclusions

In this study, we demonstrated that encapsulation of the small-molecule MAO B inhibitor, compound **1**, within NLCs enhances its ability to suppress ROS production compared to that of the free molecule. In vitro investigations employing an artificial BBB model further indicate that the nanoformulation facilitates efficient translocation of the active compound **1** across the barrier while preserving its structural and functional integrity.

Overall, these results highlight the potential of the NLC platform to improve both the bioactivity and brain delivery of compound **1**. The nanoformulation exhibited favorable cellular tolerance and maintained antioxidant efficacy in neuronal cells, supporting its suitability for further development as a delivery strategy targeting mechanisms involved in neurological disorders.

The findings presented here establish a solid experimental foundation for subsequent preclinical development. Future studies will focus on evaluating systemic administration potential, including stability and biocompatibility under physiologically relevant conditions, as well as pharmacokinetic profiling and brain biodistribution. Validation of the therapeutic performance in established models of neuroinflammation and neurodegenerative diseases will further clarify the translational potential of this approach.

Collectively, our results provide a strong proof-of-concept for the use of NLC-based nanoformulations to enhance CNS delivery of small-molecule therapeutics, supporting its potential application in NDs and its potential application in NDs.

## Supplementary Material



## Notes

RP-HPLC of compound **1**, long-term stability of **1**/NLCs by DLS, FT-IR characterization of “as-synthesized” CDs, FT-IR characterization of CDs, and assessment of the CD amount encapsulated in **1**-CD/NLCs (PDF)

## Abbreviations

Monoamine Oxidase B: (MAO B)
Nanostructured Lipid Carrier: (NLC)
Blood–Brain Barrier: (BBB)
Alzheimer’s Disease: (AD)
Parkinson’s Disease: (PD)
Neurodegenerative Disorder: (ND)
β-Amyloid: (Aβ)
Multitarget-Directed Ligand: (MTDL)
NLR family Pyrin domain containing 3: (NLRP3)
Reticuloendothelial System: (RES)
Carbon Dot: (CD)
1-Octadecene: (ODE)
1-Hexadecylamine: (HDA)
2-(N-morpholino)­ethanesulfonic acid: (MES)
Sodium chloride: (NaCl)
Disodium hydrogen phosphate: (Na_2_HPO_4_)
Potassium chloride: (KCl)
Potassium dihydrogen phosphate: (KH_2_PO_4_)
Phosphate-Buffered Saline: (PBS)
Human recombinant Monoamine Oxidase B: (MAO B)
3-(4,5-Dimethylthiazol-2-yl)-2.5-diphenyltetrazolium Bromide: (MTT)
Fluorescein Isothiocyanate-Dextran: (FITC–D)
1,2-Distearoyl-*sn*-glycero-3-phosphoethanolamine–Poly­(ethylene glycol): (DSPE-PEG)
Fetal Bovine Serum: (FBS)
Penicillin and Streptomycin: (P/S)
Dulbecco’s Modified Eagle’s Medium: (DMEM)
2′,7′-Dichlorodihydrofluorescein Diacetate: (DCFH-DA)
Dichlorofluorescein: (DCF)
Human Neuroblastoma Cells: (SH-SY5Y)
Immortalized Nontumorigenic Astrocytes: (DI TNC1)
Immortalized Brain Endothelial Cells: (bEnd-3)
American Type Culture Collection: (ATCC)
Polydispersity Index: (PDI)
Transmission Electron Microscopy: (TEM)
Attenuated Total Reflection Fourier Transform Infrared: (ATR-FTIR)
Near Infrared Region: (NIR)
Steady-State Photoluminescence: (PL)
Time-Correlated Single Photo Counting: (TCSPC)
Time-Resolved Photoluminescence: (TR-PL)
Encapsulation Efficiency: (EE%)
Drug Loading: (DL%)
Control: (CTRL)
Reactive Oxygen Species: (ROS)
Photoluminescence Intensity: (PLI)
Apparent Permeability: (Pa)
Endothelial Permeability: (Pe)
Poly­(ethylene glycol): (PEG)
3,4,5-Trimethoxyphenyl: (TMP)
P-Glycoprotein: (P-gp)
Central Nervous System: (CNS)
Food and Drug Administration: (FDA)
Low-Density Lipoprotein Receptors: (LDL).

## References

[1] La
Spada G., Miniero D. V., Rullo M., Cipolloni M., Delre P., Colliva C., Colella M., Leonetti F., Liuzzi G. M., Mangiatordi G. F. (2024). Structure-based design
of multitargeting ChEs-MAO B inhibitors based on phenyl ring bioisosteres:
AChE/BChE selectivity switch and drug-like characterization. Eur. J. Med. Chem..

[2] Castrillo, J. I. ; Oliver, S. G. Alzheimer’s as a Systems-Level Disease Involving the Interplay of Multiple Cellular Networks. In Systems Biology of Alzheimer’s Disease; Castrillo, J. I. , Oliver, S. G. , Eds.; Springer: New York, 2016; pp 3–48. 10.1007/978-1-4939-2627-5_1 26235058

[3] Bolognesi M. L. (2019). Harnessing
Polypharmacology with Medicinal Chemistry. ACS
Med. Chem. Lett..

[4] Rullo M., Catto M., Carrieri A., de Candia M., Altomare C. D., Pisani L. (2019). Chasing ChEs-MAO B
Multi-Targeting
4-Aminomethyl-7-Benzyloxy-2H-Chromen-2-ones. Molecules.

[5] Pisani L., Catto M., Leonetti F., Nicolotti O., Stefanachi A., Campagna F., Carotti A. (2011). Targeting
Monoamine
Oxidases with Multipotent Ligands: An Emerging Strategy in the Search
of New Drugs Against Neurodegenerative Diseases. Curr. Med. Chem..

[6] Pérez-Areales F. J., Turcu A. L., Barniol-Xicota M., Pont C., Pivetta D., Espargaró A., Bartolini M., De Simone A., Andrisano V., Pérez B. (2019). A novel class of multitarget
anti-Alzheimer benzohomoadamantane–chlorotacrine hybrids modulating
cholinesterases and glutamate NMDA receptors. Eur. J. Med. Chem..

[7] Reis J., Cagide F., Valencia M. E., Teixeira J., Bagetta D., Pérez C., Uriarte E., Oliveira P. J., Ortuso F., Alcaro S. (2018). Multi-target-directed ligands for Alzheimer’s
disease: Discovery of chromone-based monoamine oxidase/cholinesterase
inhibitors. Eur. J. Med. Chem..

[8] Intranuovo F., Brunetti L., DelRe P., Mangiatordi G. F., Stefanachi A., Laghezza A., Niso M., Leonetti F., Loiodice F., Ligresti A. (2023). Development
of N-(1-Adamantyl)­benzamides
as Novel Anti-Inflammatory Multitarget Agents Acting as Dual Modulators
of the Cannabinoid CB2 Receptor and Fatty Acid Amide Hydrolase. J. Med. Chem..

[9] Marques C. S., López O. ´., Bagetta D., Carreiro E. P., Petralla S., Bartolini M., Hoffmann M., Alcaro S., Monti B., Bolognesi M. L. (2020). N-1,2,3-triazole-isatin
derivatives for cholinesterase and β-amyloid aggregation inhibition:
A comprehensive bioassay study. Bioorg. Chem..

[10] Spatz P., Steinmüller S.
A. M., Tutov A., Poeta E., Morilleau A., Carles A., Deventer M. H., Hofmann J., Stove C. P., Monti B. (2023). Dual-Acting
Small Molecules:
Subtype-Selective Cannabinoid Receptor 2 Agonist/Butyrylcholinesterase
Inhibitor Hybrids Show Neuroprotection in an Alzheimer’s Disease
Mouse Model. J. Med. Chem..

[11] Rullo M., Cipolloni M., Catto M., Colliva C., Miniero D. V., Latronico T., de Candia M., Benicchi T., Linusson A., Giacchè N. (2022). Probing Fluorinated Motifs onto Dual AChE-MAO
B Inhibitors: Rational Design, Synthesis, Biological Evaluation, and
Early-ADME Studies. J. Med. Chem..

[12] Sánchez-Rodríguez R., Munari F., Angioni R., Venegas F., Agnellini A., Castro-Gil M. P., Castegna A., Luisetto R., Viola A., Canton M. (2021). Targeting monoamine oxidase to dampen NLRP3 inflammasome
activation in inflammation. Cell. Mol. Immunol..

[13] Leng F., Edison P. (2021). Neuroinflammation and
microglial activation in Alzheimer
disease: where do we go from here?. Nat. Rev.
Neurol..

[14] Carter S. F., Herholz K., Rosa-Neto P., Pellerin L., Nordberg A., Zimmer E. R. (2019). Astrocyte Biomarkers
in Alzheimer’s Disease. Trends Mol. Med..

[15] Saura J., Luque J. M., Cesura A. M., Prada M. D., Chan-Palay V., Huber G., Löffler J., Richards J. G. (1994). Increased monoamine
oxidase b activity in plaque-associated astrocytes of Alzheimer brains
revealed by quantitative enzyme radioautography. Neuroscience.

[16] Schedin-Weiss S., Inoue M., Hromadkova L., Teranishi Y., Yamamoto N. G., Wiehager B., Bogdanovic N., Winblad B., Sandebring-Matton A., Frykman S. (2017). Monoamine
oxidase B is elevated in Alzheimer disease neurons, is associated
with γ-secretase and regulates neuronal amyloid β-peptide
levels. Alzheimer’s Research and Therapy.

[17] Rullo M., La Spada G., Miniero D. V., Gottinger A., Catto M., Delre P., Mastromarino M., Latronico T., Marchese S., Mangiatordi G. F. (2023). Bioisosteric replacement based on 1,2,4-oxadiazoles in the discovery
of 1H-indazole-bearing neuroprotective MAO B inhibitors. Eur. J. Med. Chem..

[18] Pisani L., De Palma A., Giangregorio N., Miniero D. V., Pesce P., Nicolotti O., Campagna F., Altomare C. D., Catto M. (2017). Mannich base
approach to 5-methoxyisatin 3-(4-isopropylphenyl)­hydrazone: A water-soluble
prodrug for a multitarget inhibition of cholinesterases, beta-amyloid
fibrillization and oligomer-induced cytotoxicity. Eur. J. Pharm. Sci..

[19] Shafi O. (2016). Inverse relationship
between Alzheimer’s disease and cancer, and other factors contributing
to Alzheimer’s disease: a systematic review. BMC Neurol..

[20] Amiri M., Jafari S., Kurd M., Mohamadpour H., Khayati M., Ghobadinezhad F., Tavallaei O., Derakhshankhah H., Sadegh Malvajerd S., Izadi Z. (2021). Engineered Solid Lipid
Nanoparticles and Nanostructured Lipid Carriers as New Generations
of Blood–Brain Barrier Transmitters. ACS Chem. Neurosci..

[21] Das M., Sarma A., Baruah H., Basak D. (2024). Insight into central
nervous system targeted nanostructured lipid carriers via the nose
to brain pathway. RSC Pharm..

[22] Horta M., Soares P., Sarmento B., Leite Pereira C., Lima R. T. (2025). Nanostructured lipid carriers for enhanced batimastat
delivery across the blood–brain barrier: an in vitro study
for glioblastoma treatment. Drug Delivery Transl.
Res..

[23] Jeitler R., Glader C., König G., Kaplan J., Tetyczka C., Remmelgas J., Mußbacher M., Fröhlich E., Roblegg E. (2024). On the Structure, Stability,
and Cell Uptake of Nanostructured
Lipid Carriers for Drug Delivery. Mol. Pharmaceutics.

[24] Khan S. A., Qamar Z., Kamboj S., Moonis M., Rai G., Dang S., Singh P. P., Alam O., Parvez S., Baboota S. (2025). Nose to
brain delivery of nanostructured lipid carriers
loaded with rivastigmine and nilotinib for treating Alzheimer’s
disease: preparation, cell line study, and in vivo evaluation. Discover Nano.

[25] Wang L., Yang L., Liang K., Liu B., Luo Q., Wang W., Zhang D., Wang Q. (2025). Cannabidiol-Loaded
Nanostructured Lipid Carriers for Nose to Brain Delivery: An Effective
Therapeutic Approach against Epilepsy. Mol.
Pharmaceutics.

[26] Kakkar V., Mishra A. K., Chuttani K., Kaur I. P. (2013). Proof of concept
studies to confirm the delivery of curcumin loaded solid lipid nanoparticles
(C-SLNs) to brain. Int. J. Pharm..

[27] Cunha S., Costa C. P., Loureiro J. A., Alves J., Peixoto A. F., Forbes B., Sousa Lobo J. M., Silva A. C. (2020). Double Optimization
of Rivastigmine-Loaded Nanostructured Lipid Carriers (NLC) for Nose-to-Brain
Delivery Using the Quality by Design (QbD) Approach: Formulation Variables
and Instrumental Parameters. Pharmaceutics.

[28] Iacobazzi R. M., Vischio F., Arduino I., Canepa F., Laquintana V., Notarnicola M., Scavo M. P., Bianco G., Fanizza E., Lopedota A. A. (2022). Magnetic implants in vivo guiding sorafenib
liver delivery by superparamagnetic solid lipid nanoparticles. J. Colloid Interface Sci..

[29] Vischio F., Fanizza E., De Bellis V., Sibillano T., Ingrosso C., Giannini C., Laquintana V., Denora N., Agostiano A., Striccoli M. (2019). Near-Infrared Absorbing Solid Lipid Nanoparticles Encapsulating Plasmonic
Copper Sulfide Nanocrystals. J. Phys. Chem.
C.

[30] Panniello A., Di Mauro A. E., Fanizza E., Depalo N., Agostiano A., Curri M. L., Striccoli M. (2018). Luminescent
Oil-Soluble Carbon Dots
toward White Light Emission: A Spectroscopic Study. J. Phys. Chem. C.

[31] Latronico T., Rizzi F., Panniello A., Laquintana V., Arduino I., Denora N., Fanizza E., Milella S., Mastroianni C. M., Striccoli M. (2021). Luminescent PLGA Nanoparticles
for Delivery of Darunavir to the Brain and Inhibition of Matrix Metalloproteinase-9,
a Relevant Therapeutic Target of HIV-Associated Neurological Disorders. ACS Chem. Neurosci..

[32] Arduino I., Depalo N., Re F., Dal Magro R., Panniello A., Margiotta N., Fanizza E., Lopalco A., Laquintana V., Cutrignelli A. (2020). PEGylated solid lipid
nanoparticles for brain delivery of lipophilic kiteplatin Pt­(IV) prodrugs:
An in vitro study. Int. J. Pharm..

[33] Di
Marco A., Gonzalez Paz O., Fini I., Vignone D., Cellucci A., Battista M. R., Auciello G., Orsatti L., Zini M., Monteagudo E. (2019). Application of an in
Vitro Blood–Brain Barrier Model in the Selection of Experimental
Drug Candidates for the Treatment of Huntington’s Disease. Mol. Pharmaceutics.

[34] Negro R., Mastrogiacomo R., Carrieri L., Rizzi F., Arrè V., Minervini G., Fanizza E., Bianco G., Panniello A., Striccoli M. (2023). Encapsulation of MCC950 in Liposomes Decorated
with Anti-Frizzled 1 Improves Drug Bioavailability and Effectiveness
in Fatty Liver Disease. ACS Appl. Mater. Interfaces.

[35] Stark J. (1907). Ultra-violet
Fluorescence of Benzene. Nature.

[36] García-Valdivia A. A., Echenique-Errandonea E., Ramírez-Rodríguez G. B., Delgado-López J. M., Fernández B., Rojas S., Cepeda J., Rodríguez-Diéguez A. (2021). Photoluminescent
Coordination Polymers Based on Group 12 Metals and 1H-Indazole-6-Carboxylic
Acid. Inorganics.

[37] Haider M., Abdin S. M., Kamal L., Orive G. (2020). Nanostructured Lipid
Carriers for Delivery of Chemotherapeutics: A Review. Pharmaceutics.

[38] Liu Q., Guo B., Rao Z., Zhang B., Gong J. R. (2013). Strong two-photon-induced
fluorescence from photostable, biocompatible nitrogen-doped graphene
quantum dots for cellular and deep-tissue imaging. Nano Lett..

[39] Hamd-Ghadareh S., Salimi A., Fathi F., Bahrami S. (2017). An amplified comparative
fluorescence resonance energy transfer immunosensing of CA125 tumor
marker and ovarian cancer cells using green and economic carbon dots
for bio-applications in labeling, imaging and sensing. Biosens. Bioelectron..

[40] Wang L., Li B., Xu F., Li Y., Xu Z., Wei D., Feng Y., Wang Y., Jia D., Zhou Y. (2017). Visual in
vivo degradation of injectable hydrogel by real-time and non-invasive
tracking using carbon nanodots as fluorescent indicator. Biomaterials.

[41] Müller R. H., Radtke M., Wissing S. A. (2002). Solid lipid
nanoparticles (SLN) and
nanostructured lipid carriers (NLC) in cosmetic and dermatological
preparations. Adv. Drug Delivery Rev..

[42] Rainer H. M., Ranjita S., Cornelia M. K. (2011). 20 Years
of Lipid Nanoparticles (SLN
& NLC): Present State of Development & Industrial Applications. Curr. Drug Discovery Technol..

[43] Beloqui A., Solinís M. A.
´., Rodríguez-Gascón A., Almeida A. J., Préat V. (2016). Nanostructured lipid carriers: Promising
drug delivery systems for future clinics. Nanomedicine:
Nanotechnology, Biology and Medicine.

[44] de
Medeiros L. M., De Bastiani M. A., Rico E. P., Schonhofen P., Pfaffenseller B., Wollenhaupt-Aguiar B., Grun L., Barbé-Tuana F., Zimmer E. R., Castro M. A. A. (2019). Cholinergic Differentiation
of Human Neuroblastoma SH-SY5Y Cell Line and Its Potential Use as
an In vitro Model for Alzheimer’s Disease Studies. Mol. Neurobiol..

[45] Xicoy H., Wieringa B., Martens G. J. M. (2017). The SH-SY5Y cell
line in Parkinson’s
disease research: a systematic review. Mol.
Neurodegener..

[46] Mihailova L., Shalabalija D., Zimmer A., Geskovski N., Makreski P., Petrushevska M., Simonoska Crcarevska M., Glavas Dodov M. (2023). Comparative Studies of the Uptake
and Internalization
Pathways of Different Lipid Nano-Systems Intended for Brain Delivery. Pharmaceutics.

[47] Shubhra Q. T. H., Tóth J., Gyenis J., Feczkó T. (2014). Surface modification
of HSA containing magnetic PLGA nanoparticles by poloxamer to decrease
plasma protein adsorption. Colloids Surf., B.

[48] Ottenbrite, R. ; Javan, R. Biological Structures, 2005.

[49] Sonawane D., Pokharkar V. (2024). Nose to brain targeting of the donepezil nanostructured
lipid carrier in situ gel: formulation, in vitro, ex vivo, in vivo
pharmacokinetic and pharmacodynamic characterization. RSC Pharm..

[50] Devi
A R., M V. (2022). Donepezil hydrochloride loaded solid lipid nanoparticles:
formulation, in vitro−in vivo pharmacokinetic and pharmacodynamics
evaluation. Int. J. Appl. Pharm..

[51] Tekade A. R., Suryavanshi M. R., Shewale A. B., Patil V. S. (2023). Design and development
of donepezil hydrochloride loaded nanostructured lipid carriers for
efficient management of Alzheimer’s disease. Drug Dev. Ind. Pharm..

[52] Wavikar P., Pai R., Vavia P. (2017). Nose to Brain Delivery
of Rivastigmine by In Situ Gelling
Cationic Nanostructured Lipid Carriers: Enhanced Brain Distribution
and Pharmacodynamics. J. Pharm. Sci..

[53] Al
Asmari A. K., Ullah Z., Tariq M., Fatani A. (2016). Preparation,
characterization, and in vivo evaluation of intranasally administered
liposomal formulation of donepezil. Drug Des
Devel Ther.

[54] Mikitsh, J. L. ; Chacko, A.-M. Pathways for Small Molecule Delivery to the Central Nervous System across the Blood-Brain Barrier. Perspect. Med. Chem. 2014, 6, PMC.S13384. DOI: 10.4137/PMC.S13384. PMC4064947 24963272

[55] Lipinski C. A., Lombardo F., Dominy B. W., Feeney P. J. (2012). Experimental and
computational approaches to estimate solubility and permeability in
drug discovery and development settings. Adv.
Drug Delivery Rev..

[56] Pajouhesh H., Lenz G. R. (2005). Medicinal chemical
properties of successful central
nervous system drugs. NeuroRX.

[57] Kratochwil N. A., Huber W., Müller F., Kansy M., Gerber P. R. (2002). Predicting
plasma protein binding of drugs: a new approach. Biochem. Pharmacol..

[58] Fehske K. J., Müller W. E., Wollert U. (1981). The location of drug
binding sites
in human serum albumin. Biochem. Pharmacol..

[59] Achar A., Myers R., Ghosh C. (2021). Drug Delivery
Challenges in Brain
Disorders across the Blood-Brain Barrier: Novel Methods and Future
Considerations for Improved Therapy. Biomedicines.

[60] van
Assema D. M. E., Lubberink M., Boellaard R., Schuit R. C., Windhorst A. D., Scheltens P., Lammertsma A. A., van Berckel B. N. M. (2012). P-Glycoprotein Function at the Blood–Brain
Barrier: Effects of Age and Gender. Mol. Imaging
Biol..

[61] Schuster T., Mühlstein A., Yaghootfam C., Maksimenko O., Shipulo E., Gelperina S., Kreuter J., Gieselmann V., Matzner U. (2017). Potential of surfactant-coated
nanoparticles to improve
brain delivery of arylsulfatase A. J. Controlled
Release.

[62] Chen Y.-C., Hsieh W.-Y., Lee W.-F., Zeng D.-T. (2013). Effects of surface
modification of PLGA-PEG-PLGA nanoparticles on loperamide delivery
efficiency across the blood–brain barrier. J. Biomater. Appl..

[63] Luque-Michel E., Sebastian V., Larrea A., Marquina C., Blanco-Prieto M. J. (2019). Co-encapsulation
of superparamagnetic nanoparticles and doxorubicin in PLGA nanocarriers:
Development, characterization and in vitro antitumor efficacy in glioma
cells. Eur. J. Pharm. Biopharm..

[64] Gelperina S., Maksimenko O., Khalansky A., Vanchugova L., Shipulo E., Abbasova K., Berdiev R., Wohlfart S., Chepurnova N., Kreuter J. (2010). Drug delivery to the
brain using
surfactant-coated poly­(lactide-co-glycolide) nanoparticles: Influence
of the formulation parameters. Eur. J. Pharm.
Biopharm..

[65] Hoosain F. G., Choonara Y. E., Tomar L. K., Kumar P., Tyagi C., du Toit L. C., Pillay V. (2015). Bypassing
P-Glycoprotein Drug Efflux
Mechanisms: Possible Applications in Pharmacoresistant Schizophrenia
Therapy. BioMed Res. Int..

