# Supplementary material for: Nanostructured Lipid Carriers Enhance Brain Delivery and Antioxidant Efficacy of a Small-Molecule MAO B Inhibitor for Neurodegenerative Disease Therapy
Source: Mol Pharm. 2026 Apr 10;23(5):2918–34. doi: 10.1021/acs.molpharmaceut.5c01754 (PMC13147330; doi:10.1021/acs.molpharmaceut.5c01754)
Supplement: Supplementary file 1 [file mp5c01754_si_001.pdf]

Supplementary Information

Nanostructured Lipid Carriers Enhance Brain Delivery and Antioxidant Efficacy of a Small Molecule MAO B Inhibitor for Neurodegenerative Diseases Therapy

Rita Mastrogiacomo[a,b,c‡*], *Daniela Valeria Miniero*[d‡], *Mariagrazia Rullo*[e‡], *Federica Rizzi*[b,c], *Gianluca Minervini*[b], *Annamaria Panniello*[b], *Marinella Striccoli*[b,c], *Elisabetta Fanizza*[a,b,c], *Roberto Comparelli*[b,c], *Maria Lucia Curri*[a,b,c], *Marco Catto*[e], *Grazia Maria Liuzzi*[f], *Leonardo Pisani*[e], *Tiziana Latronico*[f*], *Nicoletta Depalo*[b,c*]

[a]*Department of Chemistry, University of Bari Aldo Moro, via E. Orabona 4, 70126 Bari*

[b]*Institute for Chemical and Physical Processes (IPCF)-CNR SS Bari, via Orabona 4, 70126, Bari, Italy*

[c]*National Interuniversity Consortium of Materials Science and Technology (INSTM), Bari Research Unit, 70126 Bari, Italy*

[d]*Department Medicine and Surgery, LUM University Giuseppe Degennaro, Casamassima, 70010, Italy*

[e]*Department of Pharmacy - Pharmaceutical Sciences, University of Bari Aldo Moro, 70125 Bari, Italy*

[f]*Department of Biosciences, Biotechnologies and Environment, University of Bari "Aldo Moro", 70126 Bari, Italy*

[‡]R.M., D.V.M. and M.R. contributed equally to this work

*Email: nicoletta.depalo@cnr.it; tiziana.latronico@uniba.it; rita.mastrogiacomo@uniba.it

*1. Reversed-phase high-performance liquid chromatography (RP-HPLC) of compound **1***

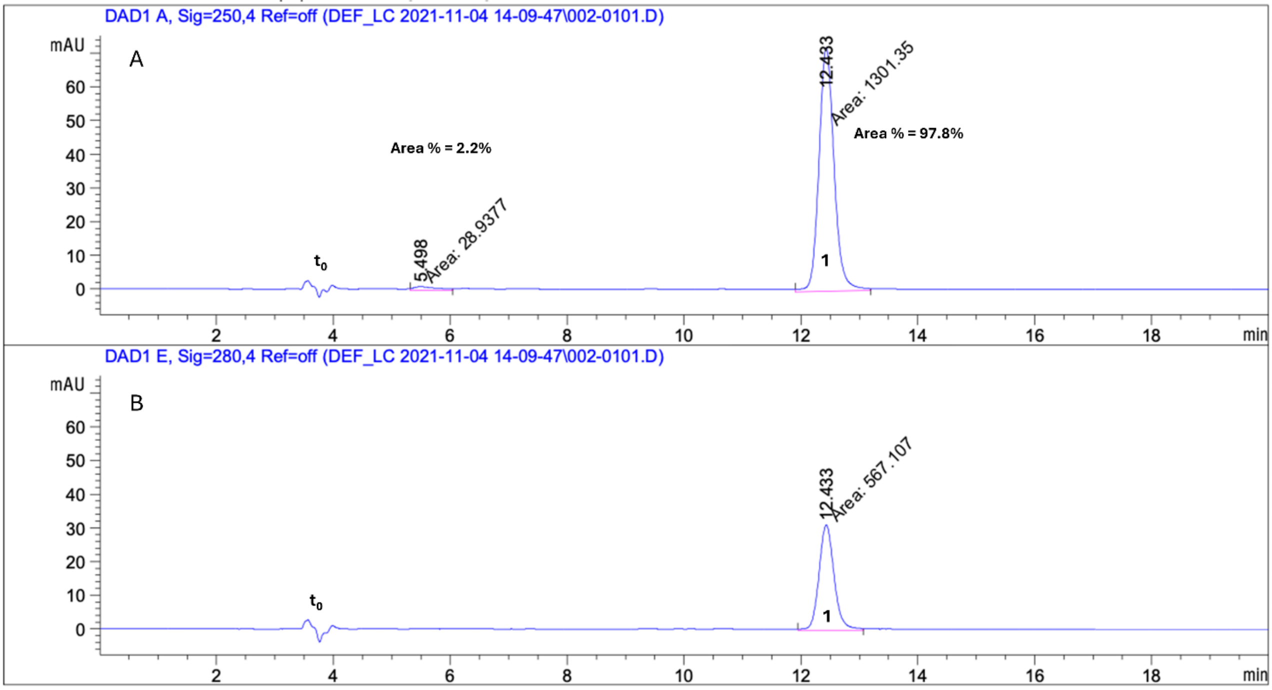

Figure S1. RP-HPLC traces of compound **1** after column chromatographic purification, before encapsulation and in vitro studies at two different wavelengths: A) 250 nm; and B) 280 nm. Samples were analysed by HPLC using a Gemini C18 4.6 mm ×150 mm, with 3 µm size particles on a Analytic Agilent 1260 Infinity multidetector system equipped with a 1200 series UV-diode array detector. UV spectra were recorded at different wavelenghts. Analytes were eluted in isocratic conditions by using a mixture of methanol and ammonium formate buffer (15 mM, pH 5.0), methanol/buffer 80%/20% (v/v), as the mobile phase. The mobile phase was filtered through a Nylon-66 membrane 0.45 µm (Supelco, USA) before use. Injection volumes were 20 µL and the flow rate was 0.5 mL/min. Data were integrated and reported using OpenLAB software (Agilent Technologies).

2. Long-Term Colloidal Stability of Nanostructured Lipid Carriers Assessed by Dynamic Light Spectroscopy (DLS)

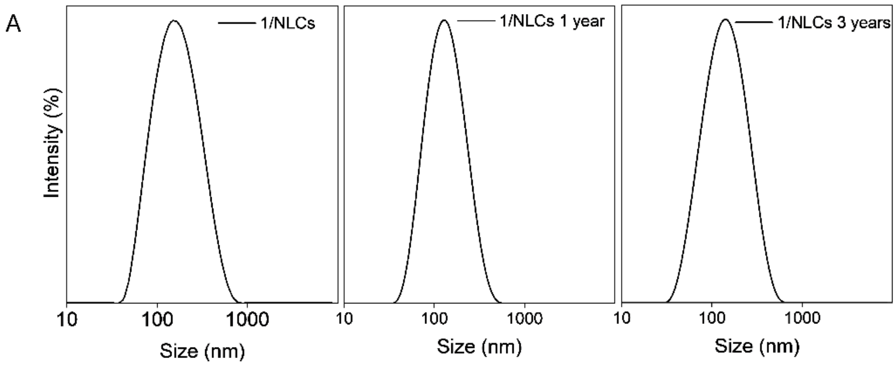

B

	size (nm)	PDI	ζ-potential (mV)
**1**/NLCs	151±3	0.217±0.013	-28.7±1.3
**1**/NLCs 1 year	146±1	0.194±0.001	-29.8±1.1
**1**/NLCs 3 years	158±1	0.220±0.002	-27.3±1.6

Figure S2. (A) Size distributions by intensity, performed by DLS measurements, and (B) summary of the size (average hydrodynamic diameter), PDI, and ζ-potential values of 1/NLCs. All reported data are presented as mean values ± SD (three technical replicates).

3. FT-IR Characterization of "as-synthesized" CDs

The FTIR spectrum of the CDs (Figure S3) shows intense absorption bands at approximately 2920 and 2850 cm^{-1}, corresponding to the asymmetric and symmetric C–H stretching vibrations of the –CH_2– and –CH_3 groups, mainly attributed to the HDA long alkyl chains coordinating the nanoparticle surface [A. Panniello et al. J Phys Chem C, 2018; D. Yi et al., ACS Applied Nano Materials, 2022]. Additional absorption features observed at ~1550 cm^{-1} and in the 1000–1100 cm^{-1} region can be ascribed to N–H bending and C=O stretching vibrations of amide bounds and to C–N/C–O stretching modes, respectively, while the sharp peak at 1690 cm−1 is attributed to the amide carbonyl stretching vibration. Such absorption signals prove the occurrence of condensation reaction between the amine groups of HDA and the carboxylic moieties of citric acid during the thermal carbonization process.

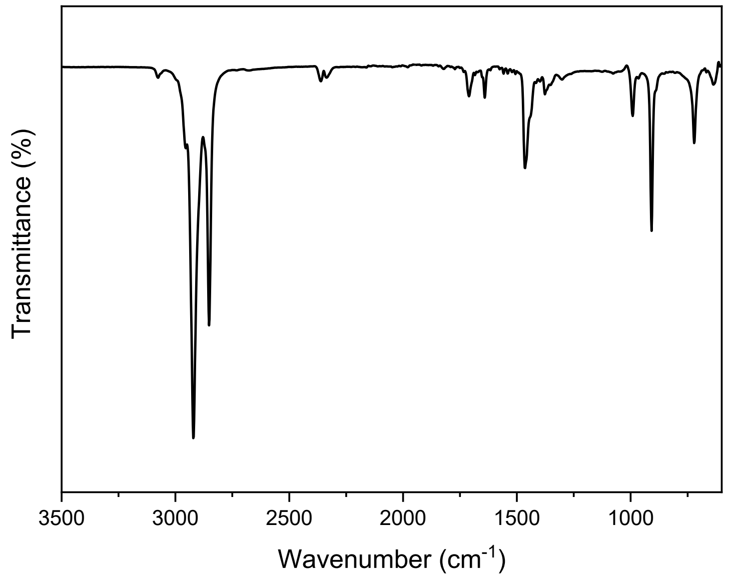

Figure S3. FT-IR spectra of the *as synthesized* CDs recorded in Attenuated Total Reflection (ATR) mode

*4. Assessment of the amount of CDs effectively encapsulated in **1**-CD/NLCs*

The compound **1** was firstly characterized by PL emission spectroscopy to determine not only its amount effectively embedded into the NLCs but also its amount passing through the artificial *in vitro* model of BBB. Chloroform solutions of the compound **1** were prepared at concentrations ranging from 0.001 μM to 5 μM. By exciting at 250 nm, the steady-state photoluminescence (SS PL) spectra in Figure S4 were acquired and the intensity of the maximum emission peak (350 nm) was used to obtain the calibration curve of the compound **1**.

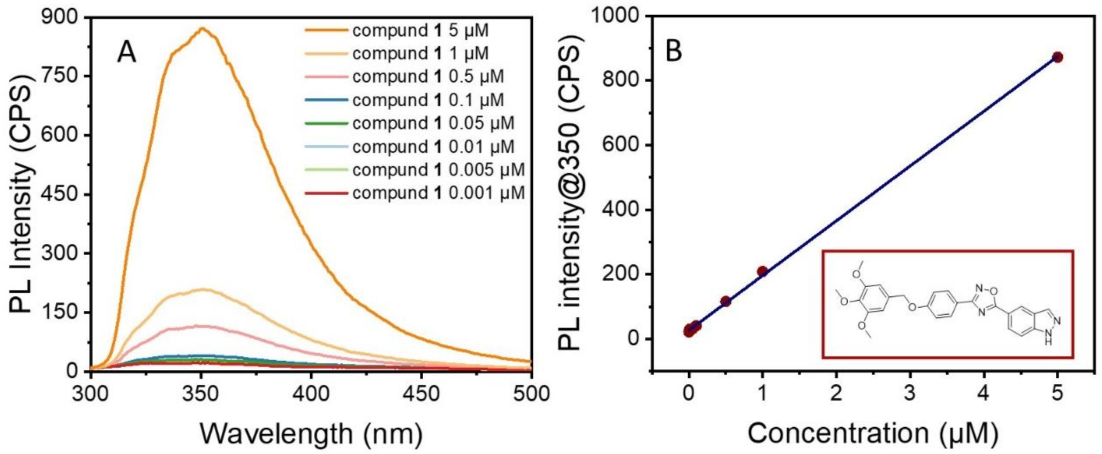

Figure S4. A) PL emission spectra of chloroform solutions of compound **1** at concentrations spanning from 0.001 μM to 5 μM and (B) respective calibration curve.
